# Decomposing Additive Genetic Variance Revealed Novel Insights into Trait Evolution in Synthetic Hexaploid Wheat

**DOI:** 10.3389/fgene.2018.00027

**Published:** 2018-02-06

**Authors:** Abdulqader Jighly, Reem Joukhadar, Sukhwinder Singh, Francis C. Ogbonnaya

**Affiliations:** ^1^Agriculture Victoria, Agriculture Research Division, AgriBio, Centre for AgriBiosciences, Bundoora, VIC, Australia; ^2^School of Applied Systems Biology, La Trobe University, Bundoora, VIC, Australia; ^3^Department of Animal, Plant and Soil Sciences, La Trobe University, Bundoora, VIC, Australia; ^4^International Maize and Wheat Improvement Center (CIMMYT), Texcoco, Mexico; ^5^Grains Research and Development Corporation, Kingston, ACT, Australia

**Keywords:** polyploidy, synthetic hexaploid wheat, diploidization, additive variance, heritability

## Abstract

Whole genome duplication (WGD) is an evolutionary phenomenon, which causes significant changes to genomic structure and trait architecture. In recent years, a number of studies decomposed the additive genetic variance explained by different sets of variants. However, they investigated diploid populations only and none of the studies examined any polyploid organism. In this research, we extended the application of this approach to polyploids, to differentiate the additive variance explained by the three subgenomes and seven sets of homoeologous chromosomes in synthetic allohexaploid wheat (SHW) to gain a better understanding of trait evolution after WGD. Our SHW population was generated by crossing improved durum parents (*Triticum turgidum;* 2n = 4x = 28, AABB subgenomes) with the progenitor species *Aegilops tauschii* (syn *Ae. squarrosa, T. tauschii*; 2n = 2x = 14, DD subgenome). The population was phenotyped for 10 fungal/nematode resistance traits as well as two abiotic stresses. We showed that the wild D subgenome dominated the additive effect and this dominance affected the A more than the B subgenome. We provide evidence that this dominance was not inflated by population structure, relatedness among individuals or by longer linkage disequilibrium blocks observed in the D subgenome within the population used for this study. The cumulative size of the three homoeologs of the seven chromosomal groups showed a weak but significant positive correlation with their cumulative explained additive variance. Furthermore, an average of 69% for each chromosomal group's cumulative additive variance came from one homoeolog that had the highest explained variance within the group across all 12 traits. We hypothesize that structural and functional changes during diploidization may explain chromosomal group relations as allopolyploids keep balanced dosage for many genes. Our results contribute to a better understanding of trait evolution mechanisms in polyploidy, which will facilitate the effective utilization of wheat wild relatives in breeding.

## Introduction

Polyploidization, whole genome duplication (WGD), is a natural process in which a single genome can be duplicated to form autopolyploids with more than two homologs for each chromosome, or multiple genomes are duplicated following hybridization between two or more species to form allopolyploids with multiple pairs of homologs derived from different ancestral genomes, termed homoeologs. Following WGD, multiple copies of duplicated genes may be lost, diverge in function, or silenced through a phenomenon called “diploidization” in which balanced dosages for many genes can be retrieved (Ohno, 1970; Lynch and Conery, 2000; Tate et al., 2009; Conant et al., 2014). Rapid genomic rearrangements and epigenetic changes have been observed directly after WGD (Ozkan et al., 2001; Shaked et al., 2001; Kashkush et al., 2002; Hegarty et al., 2008) which can cause changes in the architecture of different traits (Weiss-Schneeweiss et al., 2013).

WGD can be induced in laboratories to generate new taxa such as triticale (Stace, 1987), or to introduce new variation into known taxa such as bread wheat (*Triticum aestivum*, 2n = 6x = 42, AABBDD) which suffered a severe genetic bottleneck during its origin (Yang et al., 2009). Synthetic hexaploid wheat (SHW) can be generated by crossing *Triticum turgidum* (2n = 4x = 28, AABB) with *Aegilops tauschii* (2n = 2x = 14, DD), mimicking the natural evolutionary origin of bread wheat. SHW germplasm is a proven source of genetic diversity to improve yield (Gororo et al., 2002; Dreccer et al., 2007; Ogbonnaya et al., 2007, 2013), soil-borne pathogen (Mulki et al., 2013), insect (El-Bouhssini et al., 2013; Joukhadar et al., 2013), and fungal disease resistance (Zegeye et al., 2014; Jighly et al., 2016), as well as boron (Emebiri and Ogbonnaya, 2015) and salinity tolerance (Dreccer et al., 2004; Ogbonnaya et al., 2008a). However, it remains uncertain how the three subgenomes (A, B, and D) of bread wheat contribute to observed phenotypes or whether the wild *Aegilops* parent makes a considerable contribution to the additive genetic variance for different traits especially when crossed with an improved or elite durum wheat parent. This can be investigated by partitioning the total additive trait variance into different chromosomes in a SHW population.

Recently, a number of studies partitioned the additive variance of different traits captured by multiple sets of markers in both human and animal quantitative genetics studies. Applications varied from differentiating the variance captured by different chromosomes (Robinson et al., 2013), genotyped, and imputed variants (Lee et al., 2012), genic, and intergenic variants (Yang et al., 2011b), different SNP chips (Chen et al., 2014), to differentiating the variance of common and rare variants (Lee et al., 2013; Yang et al., 2015). In general, almost all studies reported a medium to high correlation between chromosome size and its explained additive variance for the studied traits. Yet, this approach has not been applied to any plant population, particularly among polyploid species such as wheat, where considerable efforts have gone into exploiting valuable sources of new genes from its progenitor species for cultivated wheat improvement (Ogbonnaya et al., 2013). Applying this approach to allopolyploids can provide a better understanding and a new way for differentiating the additive effects captured by different subgenomes.

In this research, we used a SHW population to investigate the contribution of each subgenome to trait variation. The SHW population was derived from crosses between wild *Ae. tauschii* parents and improved durum cultivars and was phenotyped for resistance to 10 different diseases and tolerance to two abiotic stresses. The same dataset was previously characterized in multiple genome-wide association studies (GWAS) for major genes associated with these different stresses (Mulki et al., 2013; Emebiri and Ogbonnaya, 2015; Jighly et al., 2016). However, the GWAS approach does not adequately provide the precise contribution of each chromosome/subgenome to the total heritability as genes identified through GWAS represent only a small proportion of the total heritability (Goldstein, 2009; Yang et al., 2017). Such information is critical to understanding trait evolution in newly synthesized allopolyploids and to efficiently utilize wild relatives in wheat breeding. In the present paper, we investigated this by partitioning the additive variance into each of the 21 SHW chromosomes. The relation between partitioned additive variance and chromosome, subgenome and chromosomal group size was also investigated. To the best of our knowledge, this is the first study to use this approach in polyploid or plant populations.

## Materials and methods

### SHW phenotyping and genotyping

The SHW population consists of 173 crosses between different *A. tauschii* accessions and elite durum cultivars (Table S1). The population was genotyped with DArTSeq—a genotyping by sequencing, (GBS) approach, developed by Diversity Array Technology, DArT, http://www.diversityarrays.com/. The full method is described in Sehgal et al. (2015). In brief, restriction enzymes were used first to reduce the complexity of the wheat genome and the *Pst1-RE* adapters were tagged with 96 barcodes. This strategy allows for multiplexing 96 samples in a single Illumina HiSeq2500 lane to generate around 0.5 million of 77 bp reads per sample. The generated FASTQ files were trimmed at Phred score 30 and further filtering steps and SNP calling were conducted using designed scripts developed by DArT P/L. Only SNPs with <20% missing data and >5% minor allele frequency were used in subsequent analyses. The SNP dataset used for the current study was previously published as a supplement in Jighly et al. (2016).

The SHW population was phenotyped for aluminum (Al) and boron (Br) tolerance, stem (Sr), yellow (Yr) and leaf (Lr) rusts, crown rot (Cr), yellow leaf spot (YLS), *septoria nodorum* leaf blotch (SNL) and *septoria nodorum* glume blotch (SNG), root lesion nematodes [*Pratylenchus neglectus* (Pn) and *Pratylenchus thornei* (Pt)] and cereal cyst nematode (CCN) resistance. Experimental details were previously described in (Ogbonnaya et al., 2008b; Emebiri and Ogbonnaya, 2015; Jighly et al., 2016). Briefly, the germplasm was screened in three replicates for the three rust diseases under field conditions. The most commercially important fungal pathotypes used for infection were 104–1,2,3,(6), (7), 11, 13 (accession number 200347) for Lr; 98–1,2,3,5,6 (accession number 781219) for Sr; and 134 E16A (021510) for Yr. Four different isolates (WAC 4302, WAC 4305, WAC 4306, and WAC 4309) were used in four replicates under greenhouse conditions for SNG and SNL. YLS was also screened in a controlled environment against isolates 03–0148, 03–0152, and 03–0053. For CCN, plants were considered resistant if they had less than five cysts per plant root while plants were considered susceptible if they had more than 30 cysts. Plants with 5–30 cysts were considered moderately resistant to moderately susceptible. The severity of Pn and the number of Pt nematodes per plant were used to infer the score of resistance by comparing the plant response to resistant and susceptible checks. Br tolerance was phenotyped by measuring root growth at the seedling stage on a filter paper soaked with boron while Al tolerance was measured using the hematoxylin staining of root apices method (Raman et al., 2010).

### Statistical analysis

We estimated 21 genetic relatedness matrices (GRMs) from SNPs located on each one of the SHW chromosomes following the method described in (Yang et al., 2010, 2011a). The variance explained by each chromosome was estimated using the genomic-relatedness-based restricted maximum likelihood (GREML) analysis by fitting all 21 GRMs simultaneously in the mixed linear model (Lee et al., 2012; Lee and van der Werf, 2016):

$$y=X\beta +\sum_{i\;=\;1}^{n}g_{i}+\varepsilon \;$$

Where *y* is a vector of phenotypes, *n* is the number of chromosomes (21 in our case), β is a vector of fixed effects, *X* is an incidence matrix that relates individuals to fixed effects and ε is a vector of random errors. *g*_*i*_ is a vector of random additive genetic effect attribute to chromosome *i*. The variance structure of phenotype is equal to:

$$V\;=\;\sum_{i\;=\;1}^{n}A_{i}\sigma_{g_{i}}^{2}+I\sigma_{e}^{2}$$

Where *A*_*i*_ is the GRM for chromosome *i*, $\sigma_{g_{i}}^{2}$ is the additive genetic variance captured by SNPs on chromosome *i, I* is an identity matrix and $\sigma_{e}^{2}$ is the error variance.

We ran the analysis twice, with and without including the first 10 principal components (PCs) as fixed effects. Including a number of PCs in the model can control for population structure in the germplasm; thus, the effect of population structure will be minimal if the model that fits PCs revealed similar results to the model that does not include PCs (Lee et al., 2012). The first 10 PCs were calculated using PLINK 1.9 (http://www.cog-genomics.org/plink/1.9/). To further investigate the effect of the correlation between different chromosomes due to shared structure among chromosomes (Lee et al., 2012; Yang et al., 2017), we calculated the conditional effect for each one based on the other 20 chromosomes. This was done by fitting 21 different models that each excluded one different GRM from the joint analysis. If the SNPs located on the excluded chromosome were correlated with SNPs on the other 20 chromosomes, the conditional effect analysis will overestimate the additive variance for the 20 chromosomes. Subtracting the conditional additive variance from the overall additive variance inferred from the full model is equal to the proportion of additive variance of the excluded chromosome that is not correlated with other chromosomes. This value can be used to investigate dependency among chromosomes and to confirm differences among subgenomes.

The D subgenome in our germplasm had very large LD blocks compared to the A and B subgenomes (Jighly et al., 2016) which may overestimate the heritability for the D subgenome (Speed et al., 2012). Thus, we repeated the analysis after randomly omitting 20% of the whole SNP dataset, omitting 20% of SNPs located on A and B subgenomes only, or omitting 50% of SNPs located on D subgenome. The three analyses showed similar results thus only results of the first analysis is presented in the present paper. The idea is that if we do not have enough SNP density to cover all LD blocks in both A and B subgenomes, omitting a considerable proportion of the SNPs will mask the variance captured by the deleted SNPs while keeping the D subgenome unaffected. Obtaining the same results from the original and the masked analyses suggests that each LD block is covered with adequate number of SNPs and as such, the majority of its variance can be captured with the available SNPs.

Analysis of covariance (ANCOVA) was used to determine significant differences among the three subgenomes considering (1) the subgenome size as a covariate or (2) the chromosome size as a covariate. The fitted model for the first ANCOVA analysis was: Additive Effect ~ subgenome + subgenome size. For the second analysis, we fitted the model twice, with and without including the interaction between chromosome size and subgenome. Thus, the models were: Additive Effect ~ subgenome + chromosome size; and Additive Effect ~ subgenome ^*^ chromosome size.

For each trait, a Chi-square test was performed to test whether the actual additive variance explained by the three subgenomes lies within the expected range for their values. The genome size for A, B and D subgenomes is 5727, 6274, and 4945 Mb, respectively. Thus, the expected contribution for each subgenome to the additive variance was calculated as the proportion of the subgenome size to the whole genome size, which was 33.8, 37, and 29.2% for A, B, and D subgenomes, respectively.

To further confirm that the differences among subgenomes are true and have not been inflated because of relatedness among individuals, we ran 100 replicates of the GREML analysis using randomly sampled phenotypes from the normal distribution *N* (0, 1). This analysis allows us to compare our findings to the null hypothesis given our data. True differences among subgenomes/chromosomal groups should be detected when using our empirical phenotypes and not simulated ones.

Finally, the reliability of the GREML analysis was estimated by running a 100 replicates of the analysis in which we omitted one random individual for each replicate (reduced model). Pearson correlation coefficients between additive variances of both models (full and reduced) for all chromosomes across all traits were computed. The reliability was estimated as the square of the average Pearson correlation coefficient over the 100 replicates. The reliability was used to calculate the “*attenuated correlation*” for all our correlation analyses following Charles (2005) implemented in Fisher (2014). Calculating the attenuated correlation avoids overestimating the significance of the correlation analysis by adjusting its value according to the standard deviation of our additive variance estimation.

## Results

**Figure 1:**
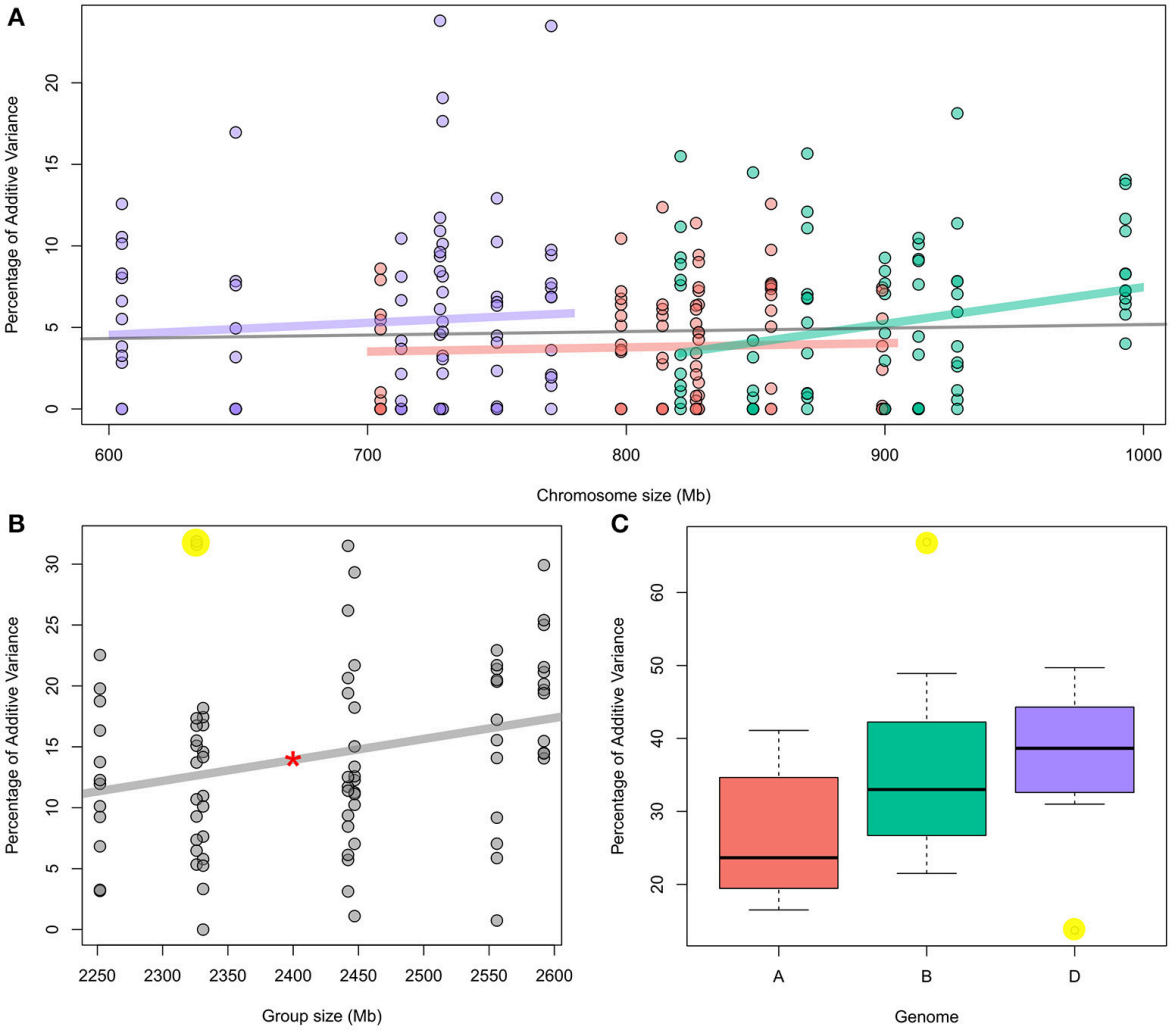
**(A)** Percentage of individual chromosome contribution to the additive variance of 12 traits as function to chromosome size; red: “A” genome chromosomes; Green: “B” genome chromosomes; and Purple: “D” genome chromosomes. The gray line represents the correlation for all 21 chromosomes. For individual traits, see Figure S1. **(B)** Percentage of each chromosomal group (seven groups) contribution to the additive variance of 12 traits as function to chromosome size. Red star over the correlation line represents its significance at *P* < 0.05. For individual traits, see Figure S2. **(C)** Boxplot showing the contribution of each genome to the additive variance of 12 traits. Highlighted yellow dots in b and c represent the outliers. For detail information, see Table 1.

The SHW dataset included 6,176 GBS based SNPs with missing data <20% and minor allele frequency >5%. The total heritability values ranged from 44.8 to 60.5% for resistance to Sr and SNG, respectively, (Table 1) with an average value of 50.4%. All estimated heritabilities were significantly higher than the heritability obtained under the null model with simulated phenotypes, which had an average of 22 and 95% confidence interval between 16.3 and 27.7%. However, it is worth noting that these values should be less than the actual heritabilities as they depend on the genotyped SNPs only (Manolio et al., 2009). The numbers presented in Table 1 represent the proportion of the total additive variance explained by each chromosome, which sum to 100 for each trait, in which negative values were recorded as zeroes (Plotted in Figure 1). The original estimations and their standard deviations can be found in Table S2. The average standard deviation across chromosomes and traits was equal to 0.077 while the reliability of the GREML analysis given the standard deviation was equal to 0.45 (0.67^2^). The considerably low reliability is a result of small population size and relatedness among individuals.

**Table 1:** The additive variance for different traits and its partitioning (as percentage of the total heritability) into different chromosomes, chromosomal groups, and genomes.

**Chr**	**Size (Mb)**	**Al**	**Br**	**CCN**	**Cr**	**Lr**	**Pn**	**Pt**	**SNG**	**SNL**	**Sr**	**YLS**	**Yr**
He	16,946	50.3	51.1	46.2	49.8	49.3	49.1	48.2	60.5	49.7	44.8	54.3	51.0
1A	798	3.6	7.2	3.5	10.4	6.4	0	6.8	5.1	0	0	3.9	5.7
2A	899	0	7.3	3.8	0	7.4	0	0	0.2	5.5	0	2.4	0
3A	828	7.5	4.3	6.4	0	7.2	9.0	4.7	4.7	0.8	0	1.6	9.4
4A	856	5.0	7.0	9.8	12.6	7.4	7.6	6.0	7.7	0	1.3	7.5	0
5A	827	6.1	6.3	0	0	2.6	0.8	2.1	11.4	5.2	0	0.5	3.4
6A	705	8.6	0	0	0	4.9	0	1.0	5.5	7.9	5.8	0.5	0
7A	814	6.4	0	0	6.1	5.1	2.7	3.1	5.7	0	12.4	0	0
1B	849	0	0	0	0	0	0	0	0.7	14.5	3.2	4.2	1.1
2B	928	2.8	11.4	0	3.8	2.6	6.0	7.0	0.6	7.8	18.1	7.8	1.1
3B	993	7.2	8.3	11.7	6.4	7.3	6.8	13.8	10.9	5.8	14.0	8.3	4.0
4B	821	1.4	0.4	0	11.2	3.3	7.9	8.9	1.1	9.3	15.5	7.6	2.2
5B	870	0.9	0.7	6.8	1.0	5.3	11.1	0	3.4	7.0	6.8	12.1	15.7
6B	913	9.1	10.5	7.6	3.3	4.4	10.1	0	9.2	0	0	0	0
7B	900	0	3.0	8.5	0	4.6	7.1	0	0	0	9.3	0	7.7
1D	605	10.1	12.6	6.6	8.3	2.8	3.3	5.5	10.5	8.0	0	3.8	0
2D	729	17.6	3.1	19.1	5.3	7.2	8.1	0	0	2.2	3.3	10.1	4.7
3D	771	6.9	1.9	2.1	23.5	0	3.6	6.9	9.4	7.4	1.4	9.8	7.7
4D	649	0	0	7.6	7.8	0	0	17.0	4.9	0	0	0	3.2
5D	750	0	4.1	6.6	0.1	2.3	6.3	12.9	6.9	0	4.5	0	10.2
6D	713	0.5	3.7	0	0	8.1	0	4.2	2.1	6.7	0	10.5	0
7D	728	6.1	8.4	0	0	10.9	9.6	0	0	11.7	4.6	9.4	23.8
Group1	2,252	13.8	19.8	10.1	18.7	9.2	3.3	12.3	16.3	22.5	3.2	12.0	6.8
Group2	2,556	20.5	21.7	22.9	9.2	17.2	14.1	7.0	0.7	15.5	21.4	20.4	5.9
Group3	2,592	21.5	14.5	20.2	29.9	14.5	19.4	25.4	25.0	14.1	15.5	19.7	21.1
Group4	2,326	6.5	7.4	17.3	31.6	10.7	15.5	31.9	13.7	9.3	16.7	15.1	5.3
Group5	2,447	7.0	11.1	13.4	1.1	10.2	18.2	15.0	21.7	12.3	11.3	12.6	29.3
Group6	2,331	18.2	14.2	7.6	3.3	17.4	10.1	5.2	16.8	14.6	5.8	11.0	0
Group7	2,442	12.5	11.4	8.5	6.1	20.6	19.4	3.1	5.7	11.7	26.2	9.4	31.5
A	5,727	37.2	32.2	23.5	29.1	41.0	20.2	23.8	40.3	19.5	19.4	16.5	18.6
B	6,274	21.5	34.1	34.6	25.7	27.6	48.9	29.8	25.8	44.5	66.8	39.9	31.8
D	4,945	41.4	33.7	41.9	45.2	31.4	31.0	46.4	33.9	36.0	13.8	43.6	49.6
Chi test	–	0.003	NS	0.01	0.002	NS	0.009	0.001	NS	0.01	0	0	0

*Negative estimations were set to 0 in this table but detailed information can be found in Table S2. The last row represents Chi square p-value which compares the actual fractional contribution of A, B, and D subgenomes to the additive variance with the expected one which assumes the percentage of the subgenome size, 33.8, 37, and 29.2% for A, B, and D subgenomes, respectively. NS: not significant at 0.05*.

For the 21 chromosomes across all traits, we found no correlation between chromosome sizes and their explained additive variance (Figure 1A; Table 2). However, for individual traits, only Sr resistance showed a significant correlation between all 21 chromosomes and their fractional contribution to the additive variance with *p*-value = 0.04 and *r* = 0.45 (Table 2; Figure S1). The median *r* value between chromosome size and fractional additive variance for all traits was equal to 0.005. When chromosomes within each subgenome were considered, only the additive variance explained by the B subgenome chromosomes showed a significant but weak correlation with chromosome size (*p*-value = 0.02 and *r* = 0.25; Figure 1A; Table 2). Neither the Sr correlation nor the B subgenome correlation were significant after adjusting them for attenuation following Charles (2005).

**Table 2:** Pearson correlation coefficient (*r* values) between the additive variance explained by all 21 chromosome sizes (column All), chromosomes within each subgenome (A, B, and D) and chromosomal group size (Groups).

**Stress**	**A**	**B**	**D**	**Groups**	**All**
SNL	−0.329	−0.435	0.007	−0.273	−0.067
SNG	−0.240	0.668	−0.283	−0.084	−0.045
YLS	0.382	−0.024	0.438	0.690	0.054
Cr	0.121	−0.049	0.119	0.002	−0.118
Lr	0.426	0.628	0.176	0.438	0.139
Sr	−0.389	0.208	0.620	0.597	0.451^*^^††^
Yr	0.006	−0.093	0.474	0.413	−0.062
CCN	0.502	0.671	−0.110	0.674	0.067
Pn	0.270	0.126	0.428	0.700	0.336
Pt	0.032	0.463	−0.223	0.004	−0.134
Br	0.683	0.764	−0.463	0.189	0.182
Al	−0.742	0.654	−0.022	0.536	−0.204
Combined	0.0407	0.25^*^	0.075	0.27^*^^††^ (0.34^**^^†^)	0.043

*The final row represents the r values considering all traits together (visualized in Figures 1A,B)*.

† *Represents the correlation coefficient after removing the two outliers in Figure 1B; this was significant at p-value < 0.05 after correcting for attenuation*.

* Significant at p-value < 0.05;

** *Significant at p-value < 0.01*.

†† *Not significant after correcting for attenuation*.

A significant correlation was evident between the cumulative size for each chromosomal group and the fractional additive variance explained by the group with *p*-value = 0.01 and *r* = 0.27 (Figure 1B, Table 2). Removing two outliers (the contribution of group 4 for Cr and Pt resistance which are highlighted in yellow, Figure 1B) strengthened this correlation with *p*-value = 0.001 and *r* = 0.34. However, when correcting the correlation for attenuation, it was significant only after removing the two outliers with *p*-value = 0.037 and *r* = 0.23. A single chromosome with the highest contribution within each group can explain about 69% of the total group additive variance on average across all traits. The relationship between fractional additive variance and the chromosomal group cumulative size for individual traits had a median value of 0.43 (Table 2) and is plotted in Figure S2.

The cumulative fractional additive variance significantly varied between the three subgenomes. The median values for the percentage of additive variance contributed by A, B, and D subgenomes were 23.7, 33, and 38.7%; respectively (Figure 1C). These values changed to 23.8, 31.8, and 41.3%, respectively, after omitting stem rust resistance, an outlier compared to other traits. ANCOVA analysis that considered the genome size as a covariate confirmed the significant differences among the three subgenomes across all 12 traits with *p*-values = 0.01. This was the only significant component in the model. The ANCOVA analysis that considered the size of chromosomes as a covariate had a *p*-value of 0.006 (same value with and without including the interaction between genome and chromosome size in the model) which was the only significant component in both models.

For individual traits, Chi-square tests showed significant differences between the actual and the expected subgenome contribution to all traits except for Br, Lr, and SNG. For Al, CCN, Cr, Pt, and Yr, only the contribution of the D subgenome was higher than expected, while the contributions of the B and D subgenomes were higher than expected for Pn, SNL, and YLS (Table 1). Br, Lr, and SNG resistances were not significantly different from the expected contribution, but the actual contribution of the D subgenome for all of them was slightly higher than expected (Table 1).

Population structure, linkage disequilibrium, and relatedness among individuals did not have an effect on our results. The inclusion of the first 10 principal components as covariates in the model did not have a large effect on heritability estimates (data not shown) which means that population structure has minimal effect on the heritability estimations. Similarly, further analysis with a randomly chosen subset of SNPs did not affect the results either (Table S3), indicating that the extended linkage disequilibrium observed in the D subgenome in this population did not overestimate the contribution of the D subgenome. Furthermore, under the null hypothesis using simulated phenotypes, the cumulative additive variance was 0.0698 (±0.026), 0.0735 (±0.027) and 0.0766 (±0.029) for the A, B, and D subgenomes, respectively, indicating true differences among subgenomes observed with empirical phenotypes that are not affected by relatedness among individuals.

**Figure 2:**
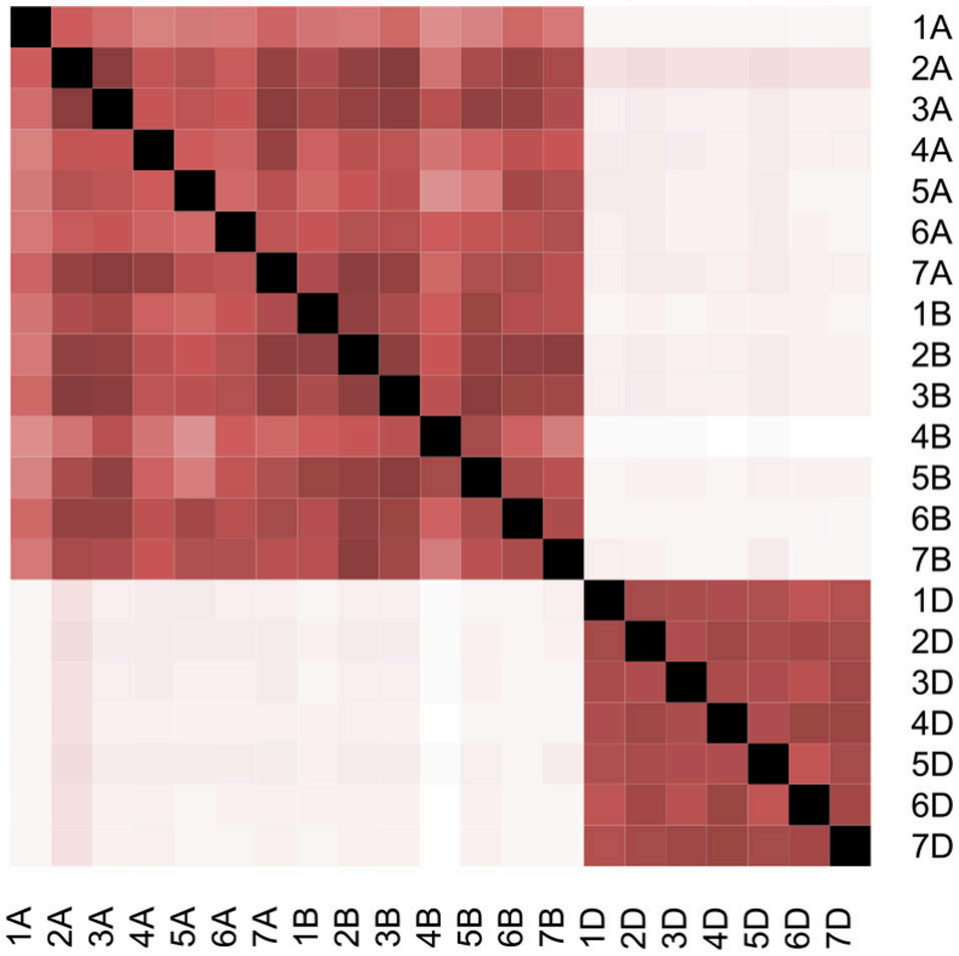
Pairwise correlation between all 21 GRMs for wheat chromosomes. White color represents Pearson coefficient = 0, while black color represents Pearson coefficient = 1.

Estimating the conditional effect for each chromosome based on the other 20 chromosomes showed considerable correlation among chromosomes (Table 3; Table S2). On average for all chromosomes across all traits, 46% of chromosome additive variance can be explained by other chromosomes. This value ranged from 20.6% for Yr resistance to 57.3% for Br tolerance (Inferred from Table 3). Interestingly, even for the conditional analysis after excluding correlated additive variances, our conclusion that the D genome had the highest contribution to the total heritability did not change with 22.3, 31.9, and 44.8% of the total additive variance attributed to the A, B, and D subgenomes, respectively. Removing Sr increased the D subgenome contribution to 45.7% and reduced the B subgenome contribution to 30.1%. The correlation among all 21 GRMs also support these results (Figure 2). All GRMs for the A and B subgenome chromosomes clustered together while GRMs for D subgenome chromosomes formed another cluster. Thus, the correlated additive variance can be explained by the same ancestor supporting the superiority of the D subgenome regardless of the low reliability of the GREML analysis.

**Table 3:** The heritability estimation using the conditional effect model (excluding the GRM of one chromosome).

**Trait**	**Al**	**Br**	**CCN**	**Cr**	**Lr**	**Pn**	**Pt**	**SNG**	**SNL**	**Sr**	**YLS**	**Yr**
He	0.503	0.511	0.462	0.498	0.493	0.491	0.482	0.605	0.497	0.448	0.543	0.510
1A	0.49 (0.013)	0.507 (0.004)	0.455 (0.007)	0.472 (0.027)	0.48 (0.013)	0.49 (0.001)	0.469 (0.013)	0.592 (0.013)	0.508 (−0.011)	0.462 (−0.014)	0.544 (−0.001)	0.486 (0.024)
2A	0.491 (0.012)	0.504 (0.007)	0.453 (0.009)	0.489 (0.009)	0.475 (0.018)	0.485 (0.007)	0.484 (−0.002)	0.607 (−0.002)	0.49 (0.007)	0.461 (−0.013)	0.541 (0.002)	0.515 (−0.005)
3A	0.486 (0.018)	0.499 (0.012)	0.451 (0.011)	0.491 (0.007)	0.481 (0.012)	0.474 (0.017)	0.477 (0.005)	0.597 (0.008)	0.499 (−0.002)	0.446 (0.002)	0.543 (0.001)	0.475 (0.035)
4A	0.487 (0.016)	0.506 (0.005)	0.432 (0.03)	0.465 (0.034)	0.484 (0.01)	0.478 (0.013)	0.476 (0.006)	0.589 (0.016)	0.492 (0.005)	0.448 (0)	0.528 (0.016)	0.512 (−0.002)
5A	0.494 (0.009)	0.496 (0.015)	0.464 (−0.002)	0.504 (−0.006)	0.496 (−0.003)	0.49 (0.001)	0.479 (0.003)	0.586 (0.02)	0.49 (0.007)	0.455 (−0.007)	0.545 (−0.001)	0.502 (0.008)
6A	0.483 (0.02)	0.499 (0.012)	0.458 (0.004)	0.49 (0.008)	0.484 (0.009)	0.487 (0.004)	0.487 (−0.005)	0.593 (0.012)	0.485 (0.012)	0.433 (0.015)	0.552 (−0.009)	0.51 (0)
7A	0.487 (0.016)	0.474 (0.037)	0.451 (0.011)	0.49 (0.008)	0.485 (0.008)	0.482 (0.009)	0.476 (0.006)	0.599 (0.006)	0.496 (0.001)	0.426 (0.022)	0.537 (0.006)	0.505 (0.005)
1B	0.519 (−0.016)	0.502 (0.009)	0.471 (−0.009)	0.508 (−0.01)	0.489 (0.005)	0.481 (0.01)	0.494 (−0.012)	0.612 (−0.007)	0.471 (0.026)	0.451 (−0.003)	0.544 (−0.001)	0.505 (0.005)
2B	0.496 (0.007)	0.463 (0.048)	0.445 (0.017)	0.492 (0.006)	0.491 (0.002)	0.474 (0.017)	0.478 (0.004)	0.607 (−0.002)	0.48 (0.017)	0.408 (0.04)	0.517 (0.026)	0.508 (0.002)
3B	0.483 (0.02)	0.51 (0.001)	0.431 (0.031)	0.47 (0.028)	0.474 (0.019)	0.483 (0.008)	0.458 (0.024)	0.585 (0.02)	0.48 (0.017)	0.423 (0.025)	0.526 (0.018)	0.509 (0.001)
4B	0.51 (−0.007)	0.514 (−0.003)	0.473 (−0.011)	0.437 (0.061)	0.482 (0.011)	0.472 (0.019)	0.471 (0.011)	0.605 (0)	0.473 (0.025)	0.418 (0.03)	0.523 (0.02)	0.472 (0.038)
5B	0.502 (0.001)	0.513 (−0.002)	0.455 (0.007)	0.497 (0.001)	0.48 (0.013)	0.473 (0.018)	0.487 (−0.005)	0.591 (0.014)	0.496 (0.001)	0.44 (0.008)	0.506 (0.037)	0.469 (0.042)
6B	0.471 (0.033)	0.508 (0.003)	0.443 (0.02)	0.486 (0.012)	0.487 (0.006)	0.462 (0.029)	0.484 (−0.002)	0.585 (0.02)	0.496 (0.001)	0.456 (−0.008)	0.514 (0.029)	0.5 (0.01)
7B	0.5 (0.003)	0.514 (−0.003)	0.445 (0.017)	0.493 (0.005)	0.488 (0.005)	0.487 (0.004)	0.482 (0)	0.626 (−0.021)	0.498 (−0.001)	0.431 (0.017)	0.555 (−0.012)	0.496 (0.015)
1D	0.47 (0.033)	0.52 (−0.009)	0.454 (0.008)	0.47 (0.028)	0.496 (−0.003)	0.483 (0.008)	0.475 (0.007)	0.547 (0.058)	0.48 (0.017)	0.438 (0.01)	0.532 (0.011)	0.484 (0.027)
2D	0.463 (0.04)	0.511 (0)	0.364 (0.098)	0.483 (0.015)	0.472 (0.021)	0.475 (0.017)	0.465 (0.017)	0.581 (0.024)	0.494 (0.003)	0.444 (0.004)	0.528 (0.015)	0.486 (0.024)
3D	0.49 (0.013)	0.506 (0.005)	0.467 (−0.005)	0.401 (0.097)	0.48 (0.013)	0.484 (0.007)	0.471 (0.011)	0.592 (0.013)	0.474 (0.023)	0.442 (0.006)	0.511 (0.032)	0.486 (0.024)
4D	0.504 (−0.001)	0.494 (0.017)	0.445 (0.017)	0.481 (0.017)	0.489 (0.004)	0.491 (0)	0.429 (0.053)	0.589 (0.016)	0.491 (0.006)	0.456 (−0.008)	0.534 (0.009)	0.498 (0.012)
5D	0.49 (0.013)	0.494 (0.017)	0.454 (0.008)	0.497 (0.001)	0.489 (0.004)	0.481 (0.011)	0.446 (0.036)	0.589 (0.016)	0.483 (0.014)	0.431 (0.017)	0.503 (0.04)	0.455 (0.055)
6D	0.507 (−0.004)	0.504 (0.007)	0.454 (0.008)	0.494 (0.004)	0.47 (0.023)	0.494 (−0.003)	0.473 (0.01)	0.607 (−0.002)	0.477 (0.02)	0.443 (0.005)	0.514 (0.029)	0.497 (0.013)
7D	0.491 (0.012)	0.493 (0.019)	0.457 (0.005)	0.498 (0)	0.469 (0.024)	0.47 (0.021)	0.478 (0.004)	0.589 (0.017)	0.469 (0.029)	0.443 (0.005)	0.515 (0.028)	0.445 (0.065)
A contribution%	37.3	42.2	23.4	25.3	31.8	23.5	15.7	27.5	13.9	18.9	7.8	17.8
B contribution%	22.9	28.0	29.9	30.7	27.7	47.5	18.6	19.8	37.7	58.3	40.8	27.9
D contribution%	39.8	29.8	46.8	44.0	40.5	29.0	65.7	52.7	48.5	22.8	51.4	54.3
Chi test	0.008	NS	0.001	0.005	0.03	0.05	0	0	0	0	0	0

*The values between brackets describes the additive variance inferred from the full model (the first row in the table) minus the conditional total additive variance. The last three rows represent the contribution of each subgenome to total independent additive variance (values between brackets). The last row represents Chi square p-value which compares the conditional contribution of A, B and D subgenomes to the additive variance with the expected one which assumes the percentage of the subgenome size, 33.8, 37, and 29.2% for A, B, and D subgenomes, respectively. NS, not significant at 0.05*.

## Discussion

Decomposing additive genetic variance based on different set of SNPs has become a commonly used method in quantitative genetics in recent years (Yang et al., 2010, 2011a,b, 2015; Lee et al., 2012). Researchers usually remove related individuals to ensure that they are capturing SNP-based heritability only (Yang et al., 2017). Although this is possible in human genetics and some animal populations that have large effective population size, it is impossible to have such optimal populations containing distinctly related individuals in species such as bread wheat with extremely small effective population sizes (Joukhadar et al., 2017). For this reason, the heritability estimated with this method in populations of species such as bread wheat will be a mixture of SNP-based heritability from phenotypic correlation due to unrelated individuals and pedigree-based heritability from phenotypic correlation due to relatedness (Yang et al., 2017). One advantage of using related individuals is that the analysis requires smaller populations to obtain an acceptable standard error (SE), because SE is negatively correlated with the average relatedness among individuals. Yang et al. (2017) pointed out that the SE can be further decreased if rare SNPs are excluded from the analysis.

Linkage disequilibrium (LD) can cause a huge bias for decomposing additive variance analysis as the variance estimation depends on the LD between the causal variant and the closest genotyped SNPs (Speed et al., 2012). The D subgenome in our population showed large LD blocks (Jighly et al., 2016) but this did not result in over estimating its contribution because there were sufficient SNPs to capture most additive variance in the A and B subgenomes (Table S3). This is not unexpected for populations with small effective population size like SHW. For example, randomly selecting 10K out of 354K SNPs reduced the captured additive variance by only 1% for different traits in chickens (Abdollahi-Arpanahi et al., 2014). Population structure also did not affect the estimation as the estimations were very similar to the model that involved the first 10 PCs as covariates (Lee et al., 2012), although considerable correlation between different chromosomes was observed in this germplasm (Table 3; Table S2). On the other hand, this correlation did not affect our conclusion that the D subgenome had a higher contribution to the total additive variance relative to the A and B subgenomes (Table 3; Table S2), and especially that GRMs of the D subgenome chromosomes were clustered together and were not correlated with any of the 14 GRMs of the A and B subgenome chromosomes (Figure 2).

Almost all studies that have partitioned additive variance have shown a significant correlation exists between chromosome size and variance (e.g., Yang et al., 2011b; Lee et al., 2012; Robinson et al., 2013). In the present study using SHW, however, chromosome size was not correlated with explained additive variance for any trait, although a weak correlation was observed for chromosomes within the B subgenome. The significant correlation for Sr (Table 2) cannot be attributed to chromosome size directly, but rather to differences in size between D and B subgenomes, which explained 13.8 and 66.8% of the additive variance, respectively (Figure S1; Table 1). The previous two correlations became non-significant after correcting for attenuation.

In contrast to what we found for all individual chromosomes, a significant but weak correlation was found between the cumulative sizes and cumulative additive variances for each chromosomal group (Figure 1B). In polyploids, the balanced dosage hypothesis, which involves gene loss, functional divergence and epigenetic changes in newly synthesized polyploids, has been widely discussed and has been proven for many gene families (Ohno, 1970; Lynch and Conery, 2000; Tate et al., 2009; Buggs et al., 2010, 2012; Xiong et al., 2011; Feldman and Levy, 2012; Conant et al., 2014; Dodsworth et al., 2016). We hypothesize that these structural and functional changes during diploidization keep a single functional copy for each gene in one homoeolog and thus, larger chromosomes may not necessarily have higher contribution to the additive variance if functional copies are not distributed equally in the three homoeologs. Instead, when considering the three homoeologs together, all genes will have functional copies. Thus, larger chromosomal groups may have higher contribution to the additive variance. This may explain the correlation between group size and effect. Another important finding is that one homoeolog can dominate the group additive effect within each chromosomal group with an average of 69% of the total group additive variance (Inferred from Table 1). Future research using larger populations should consider the relation between variance and chromosome size in both SHWs and their progenitors to further confirm this finding and to better understand underlying mechanisms that allow one homoeolog to dominate the group additive effect.

Pont et al. (2013) showed that the D subgenome generally dominated the tetraploid A and B subgenomes in hexaploid wheat by analyzing synteny and conserved orthologous gene data. Our results also showed this for stress resistance traits and that the dominance effect of the D subgenome was greater with regard to the A than the B subgenome with the median percentage of additive variance across all traits for A subgenome being 23.7% (Figure 1C). However, this cannot be generalized for all traits. For instance, the A subgenome contributed 9.6% more than the D subgenome to Lr resistance, whereas the B subgenome dominated the A and D subgenomes for Sr resistance (Table 1). Lagudah et al. (1993) showed that transferring Sr and Lr resistance form *Ae. tauschii* to hexaploid wheat is partially or fully suppressed by unknown mechanisms while Kerber and Green (1980) reported a suppressor for A and B subgenome Sr resistance in chromosome 7D. Later studies have indicated that suppression of the resistance of one subgenome of bread wheat by the other subgenomes is affected by SHW parents and pathogen isolates (Kema et al., 1995; Badebo et al., 1997; Ogbonnaya et al., 2013). Thus, efficient implementation of SHW in breeding programs should combine superior chromosomes within each chromosomal group for each trait independently, although the general trend showed that the D subgenome had a higher contribution to the additive variance. Future research should investigate suppression mechanisms and whether the general D subgenome superior additive contribution is a result of suppressing A and B subgenomes resistance to different biotic and abiotic stresses.

## Author contributions

AJ: suggested and planned the study, analyzed the data and drafted the manuscript; RJ: assisted with R scripting and drafted the manuscript; SS: provided the GBS data; FO: planned the study, provided the phenotypic data, drafted the manuscript and gave the final acceptance for the manuscript to be submitted; All authors read and approved the final copy of the manuscript.

### Conflict of interest statement

The authors declare that the research was conducted in the absence of any commercial or financial relationships that could be construed as a potential conflict of interest.

## Supplementary material

Figure S1 Percentage of individual chromosome contribution to the additive variance for each trait as function to chromosome size. Colors represents different subgenomes; red: “A” subgenome chromosomes; Green: “B” subgenome chromosomes; and Purple: “D” subgenome chromosomes. The gray line represents the correlation for all 21 chromosomes.

Figure S2 Percentage of chromosomal group contribution to the additive variance for each trait as function to chromosome size.

Table S1 Pedigree and passport information for the SHW population.

Table S2 The first line for each chromosome contains information about the estimated additive variance for different traits and their standard deviations, between brackets, using the full model (the model that fits 21 GRMs). The second line for each chromosome is the heritability estimation using the conditional effect model (excluding the GRM of one chromosome). Values between brackets describes the additive variance inferred from the full model (the first row in the table) minus the conditional total additive variance. The second line is exactly the same as Table 3 in the paper but was repeated here for easier comparisons between the full and the conditional models.

Table S3 The additive variance for different traits and its partitioning (as percentage of the total heritability) into different chromosomes, chromosomal groups and subgenomes for subset of the whole data set that includes 80% of our SNPs.
