# Supplementary figures and images for: Decomposing Additive Genetic Variance Revealed Novel Insights into Trait Evolution in Synthetic Hexaploid Wheat
Source: Front Genet. 2018 Feb 6;9:27. doi: 10.3389/fgene.2018.00027 (PMC5807918; doi:10.3389/fgene.2018.00027)

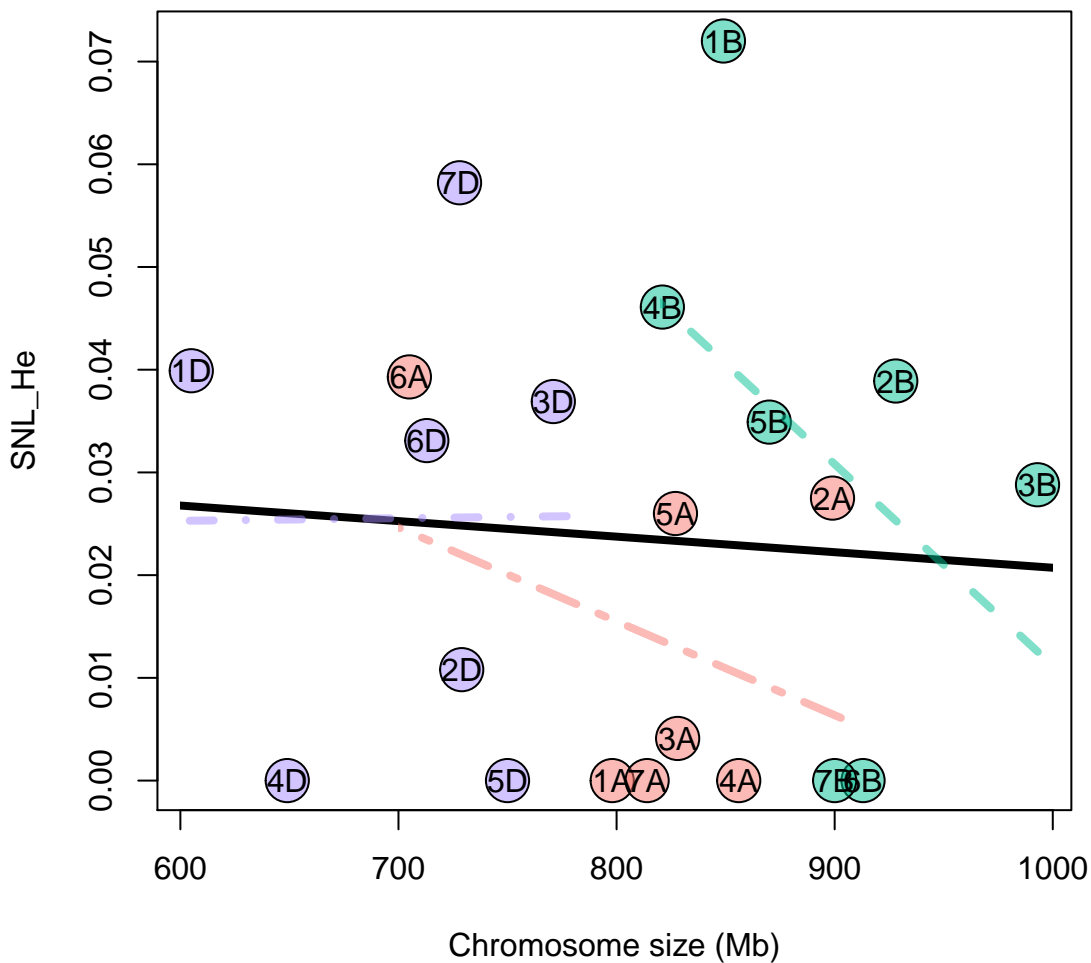

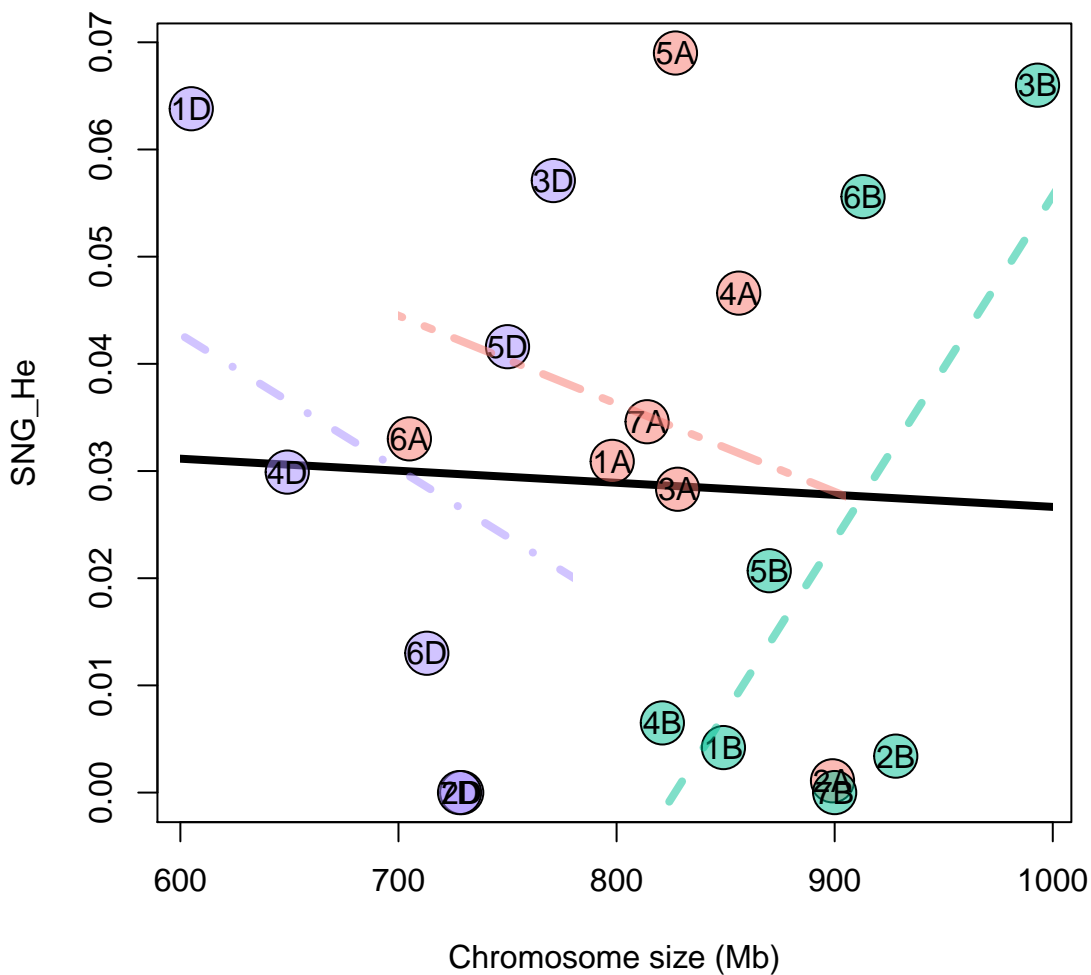

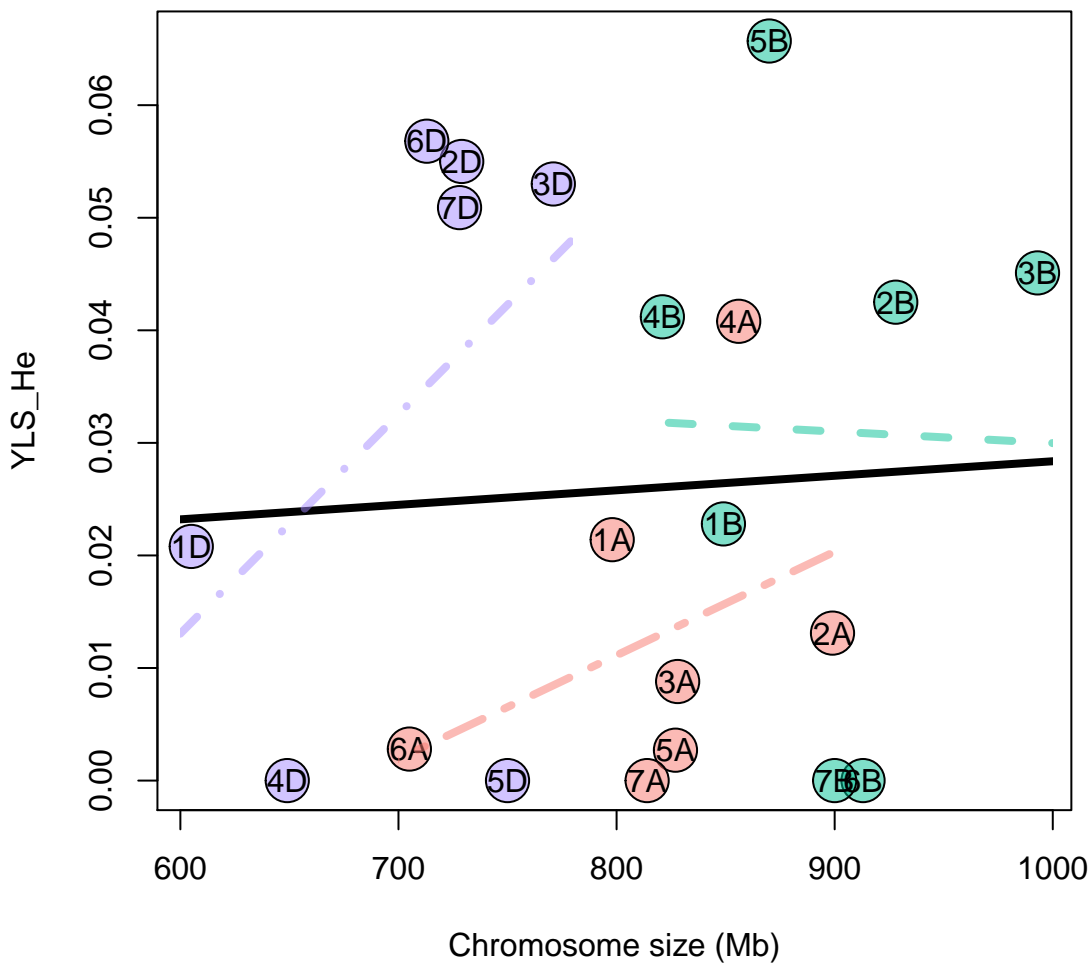

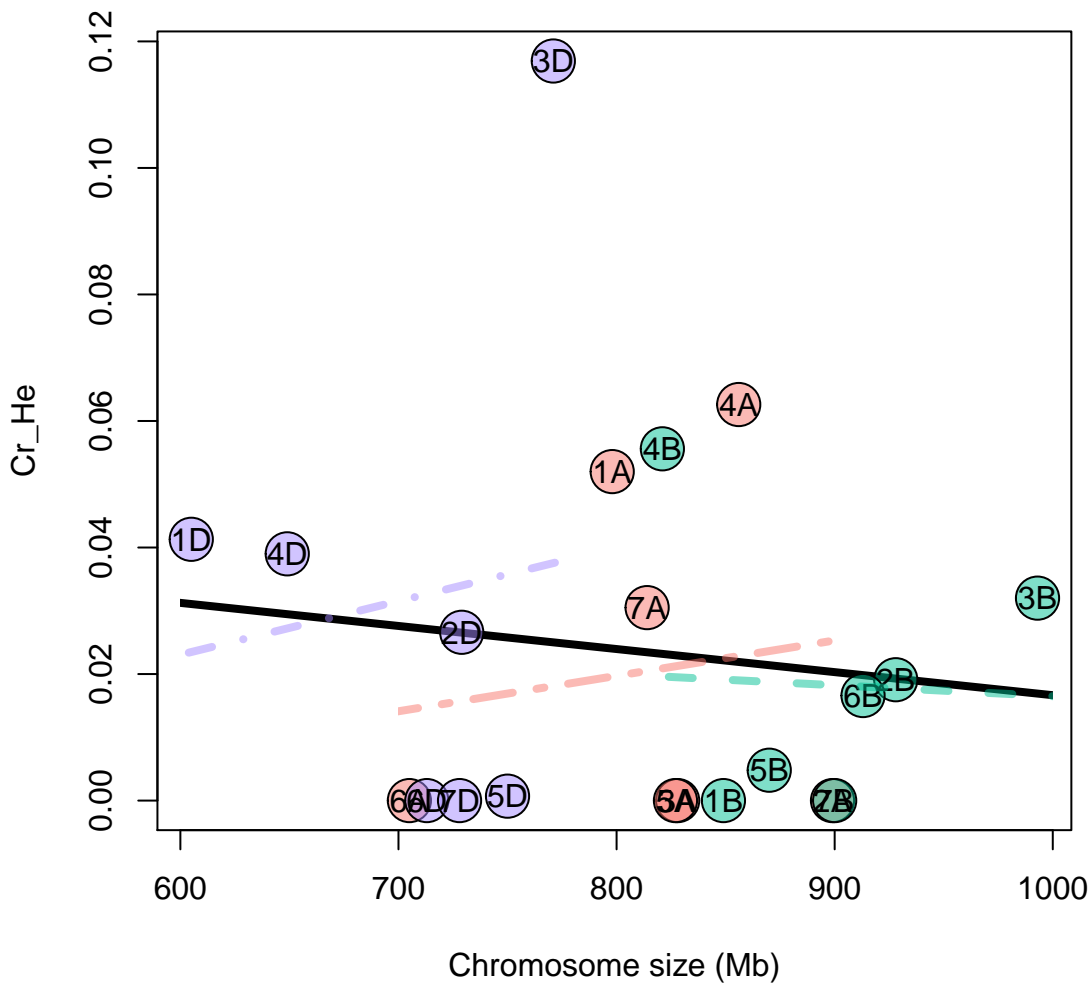

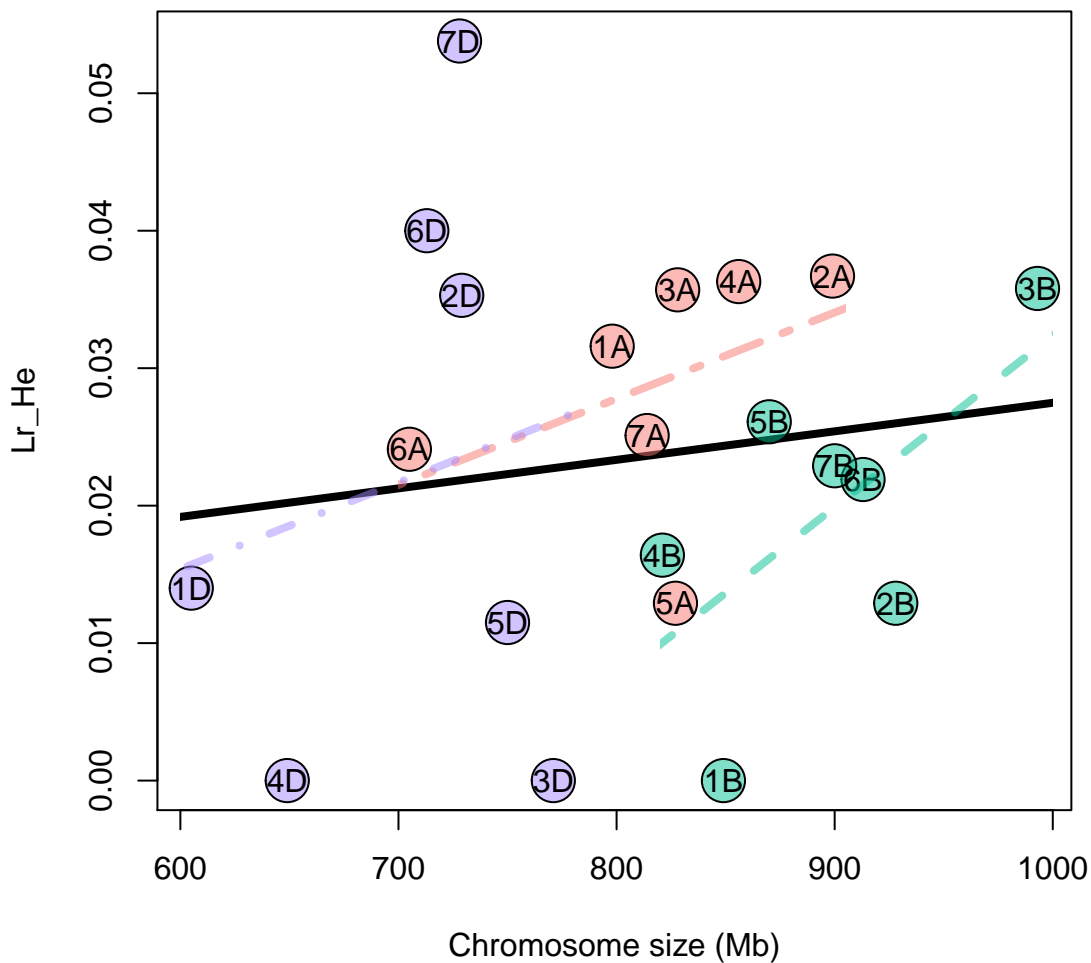

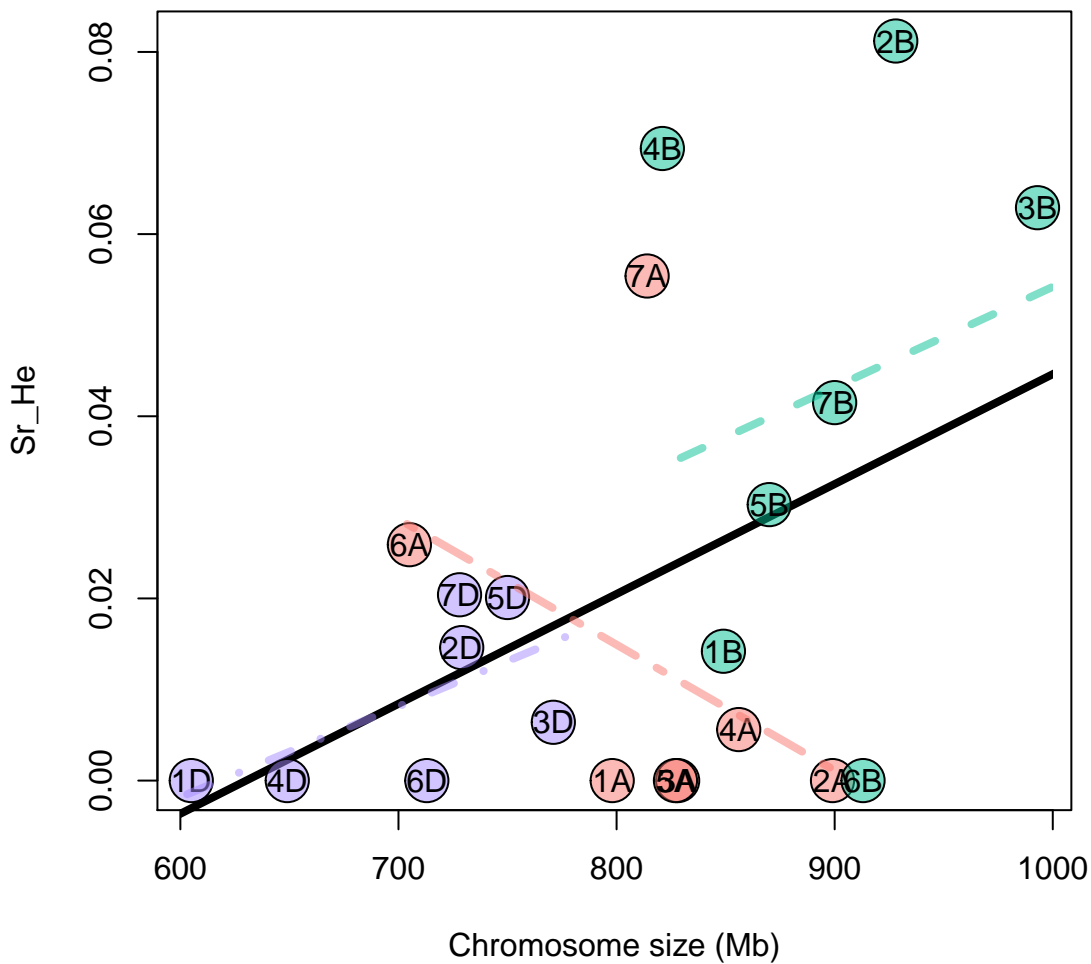

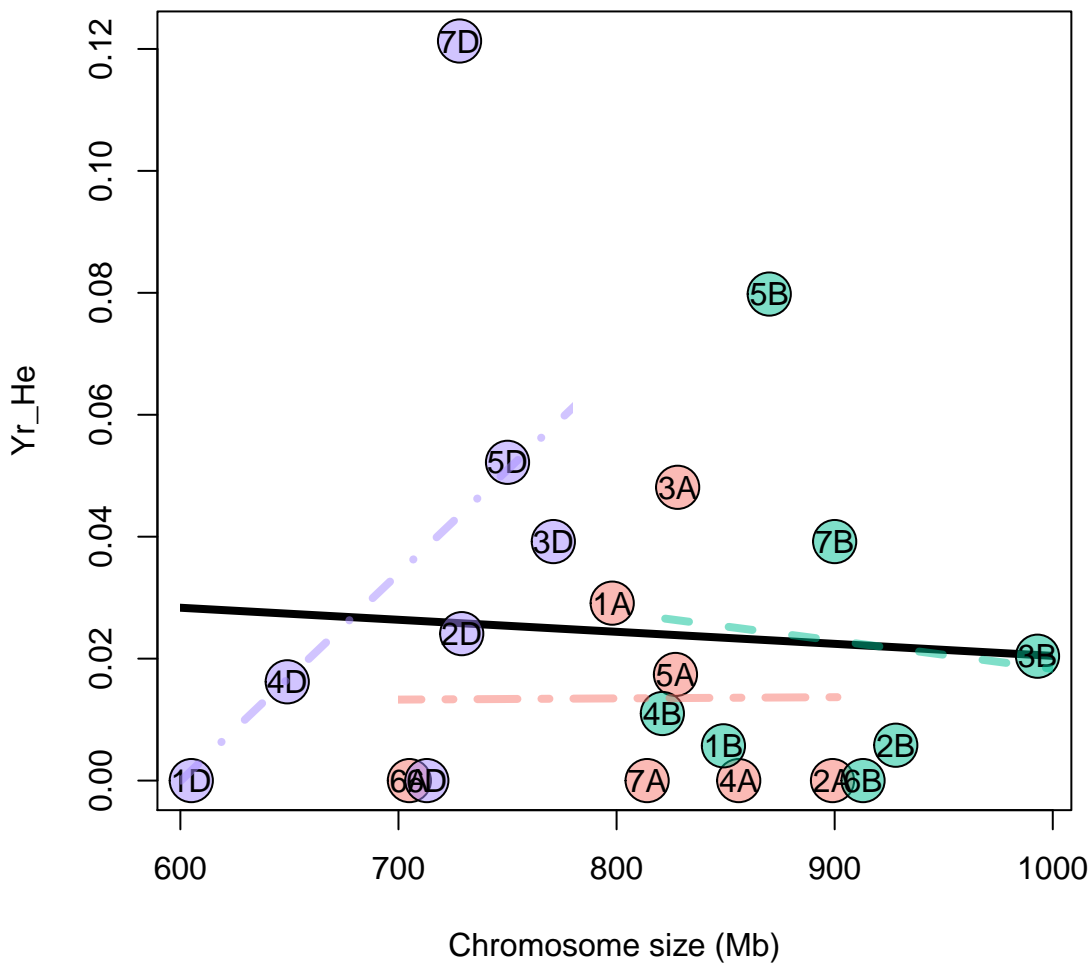

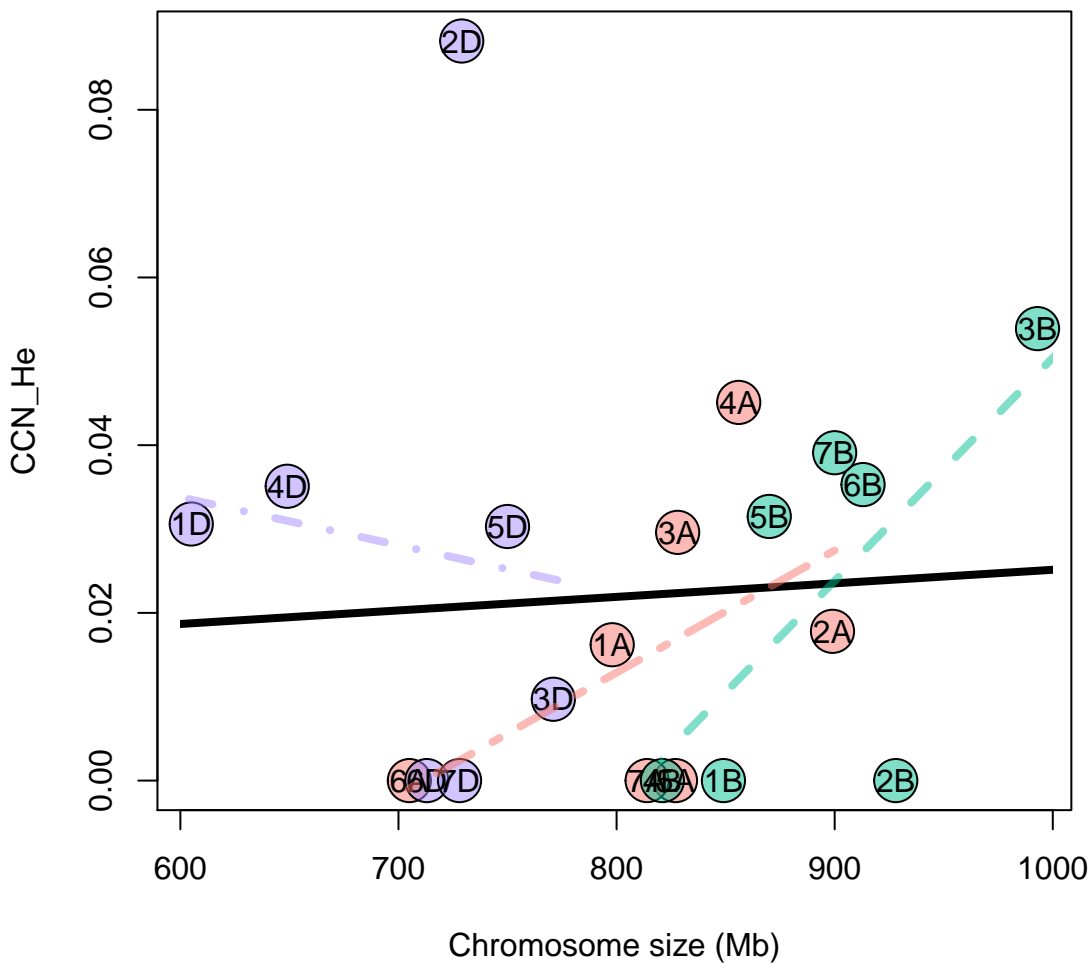

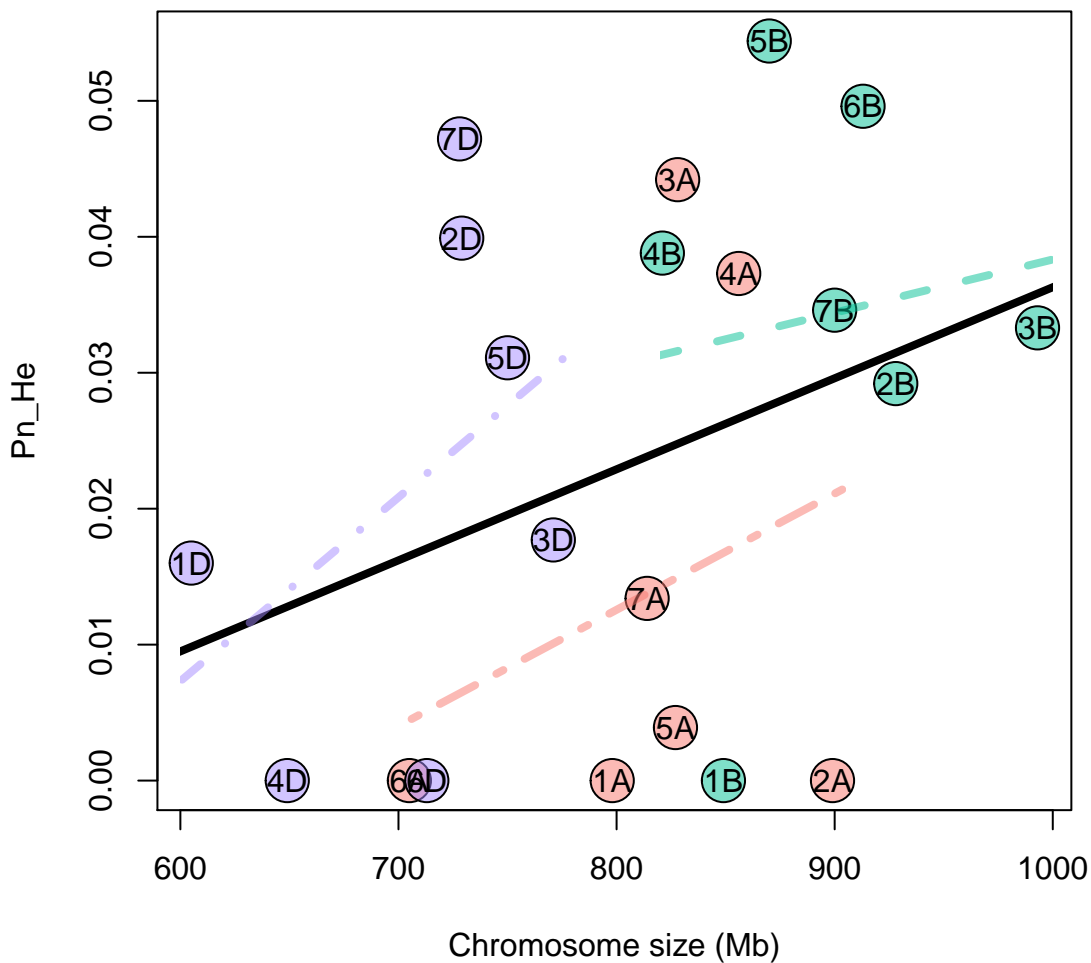

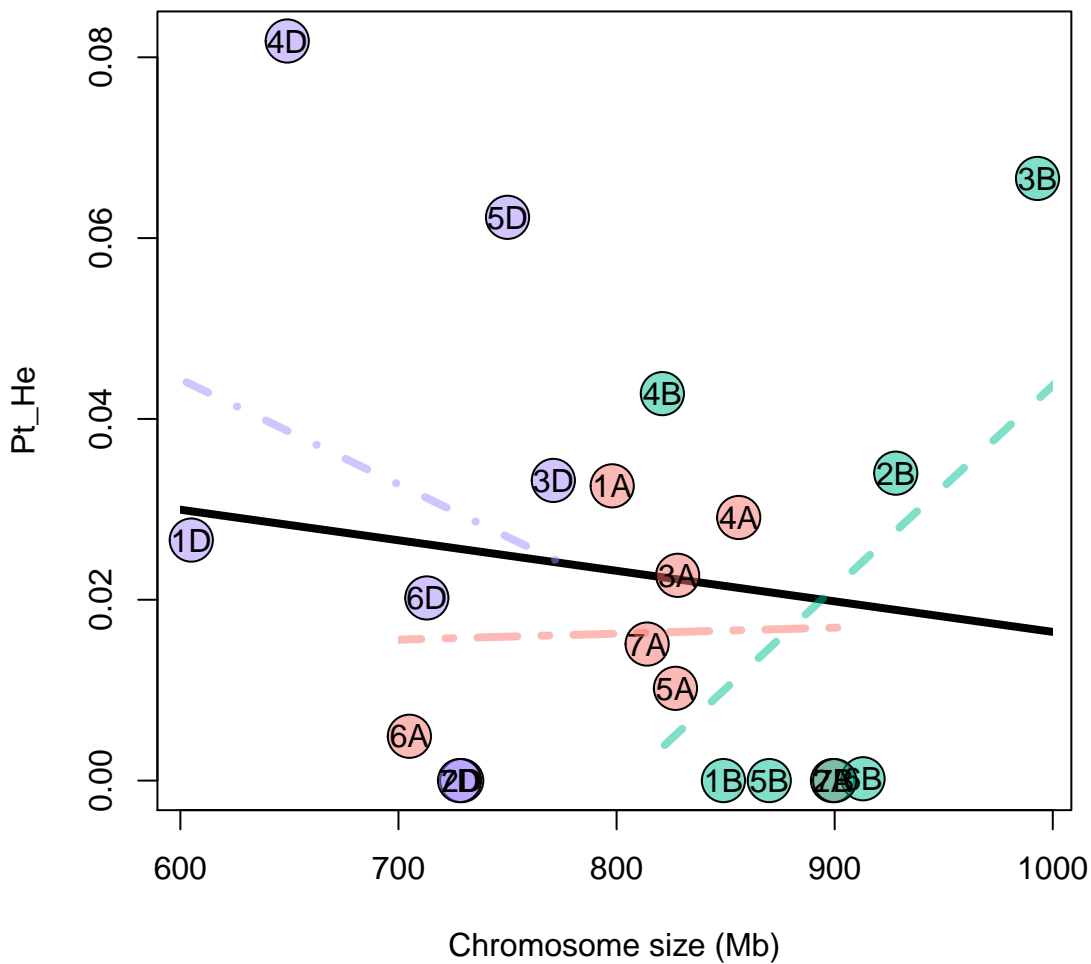

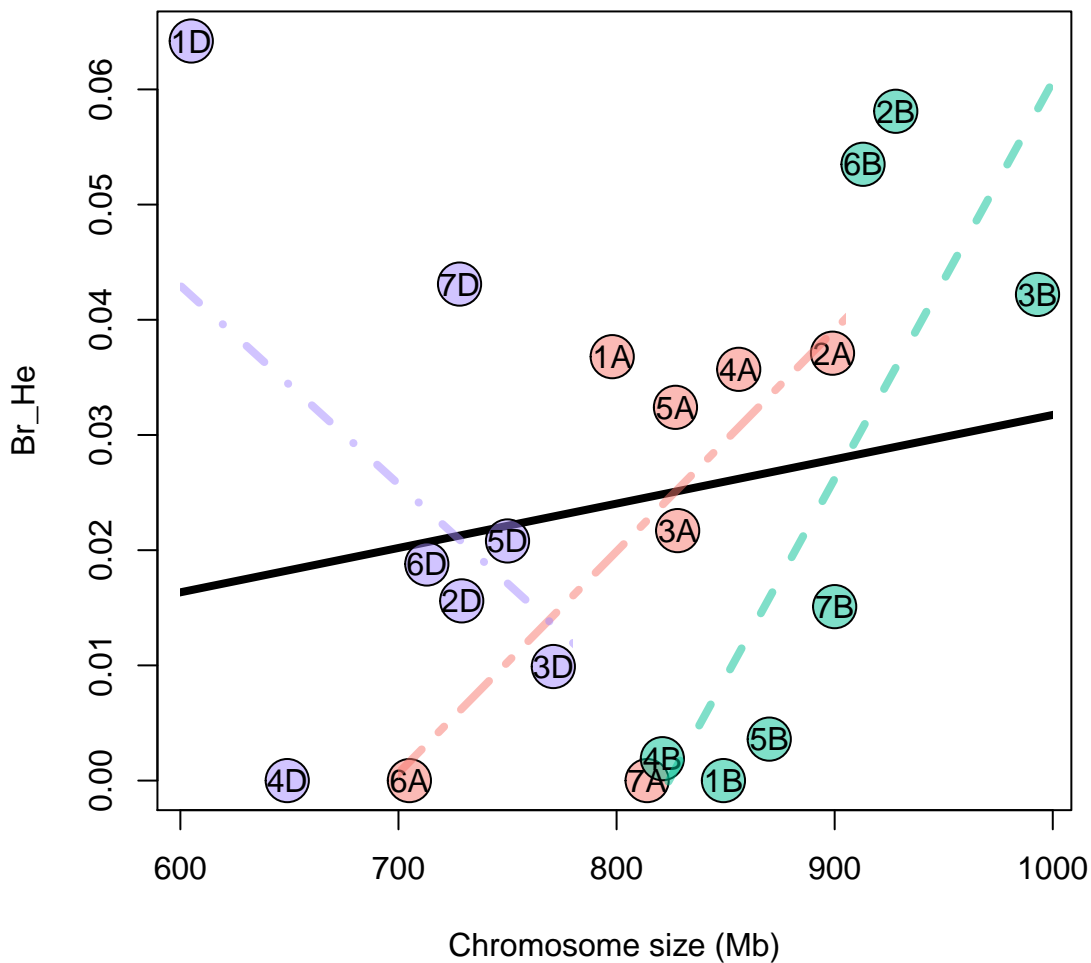

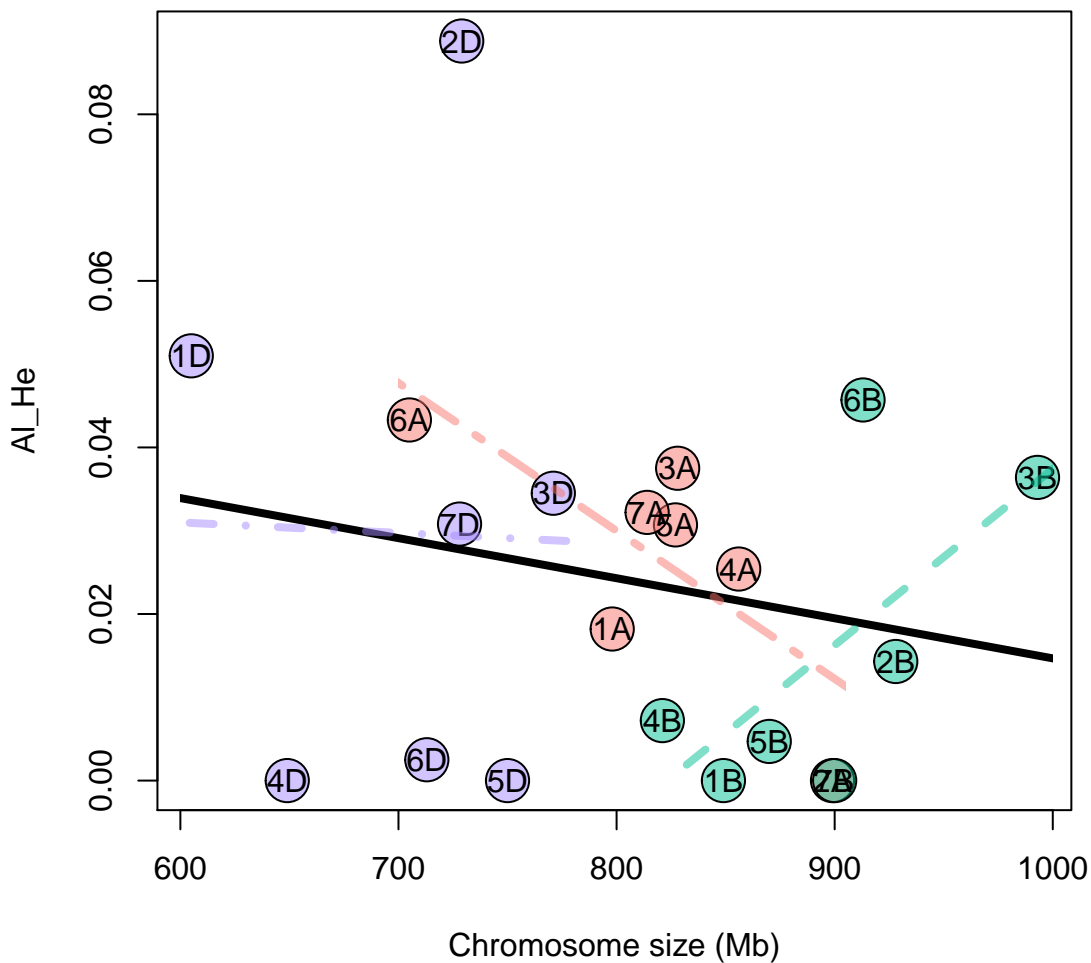

Supplement: Figure S1 — Percentage of individual chromosome contribution to the additive variance for each trait as function to chromosome size. Colors represents different subgenomes; red: “A” subgenome chromosomes; Green: “B” subgenome chromosomes; and Purple: “D” subgenome chromosomes. The gray line represents the correlation for all 21 chromosomes. [file Image1.PDF]

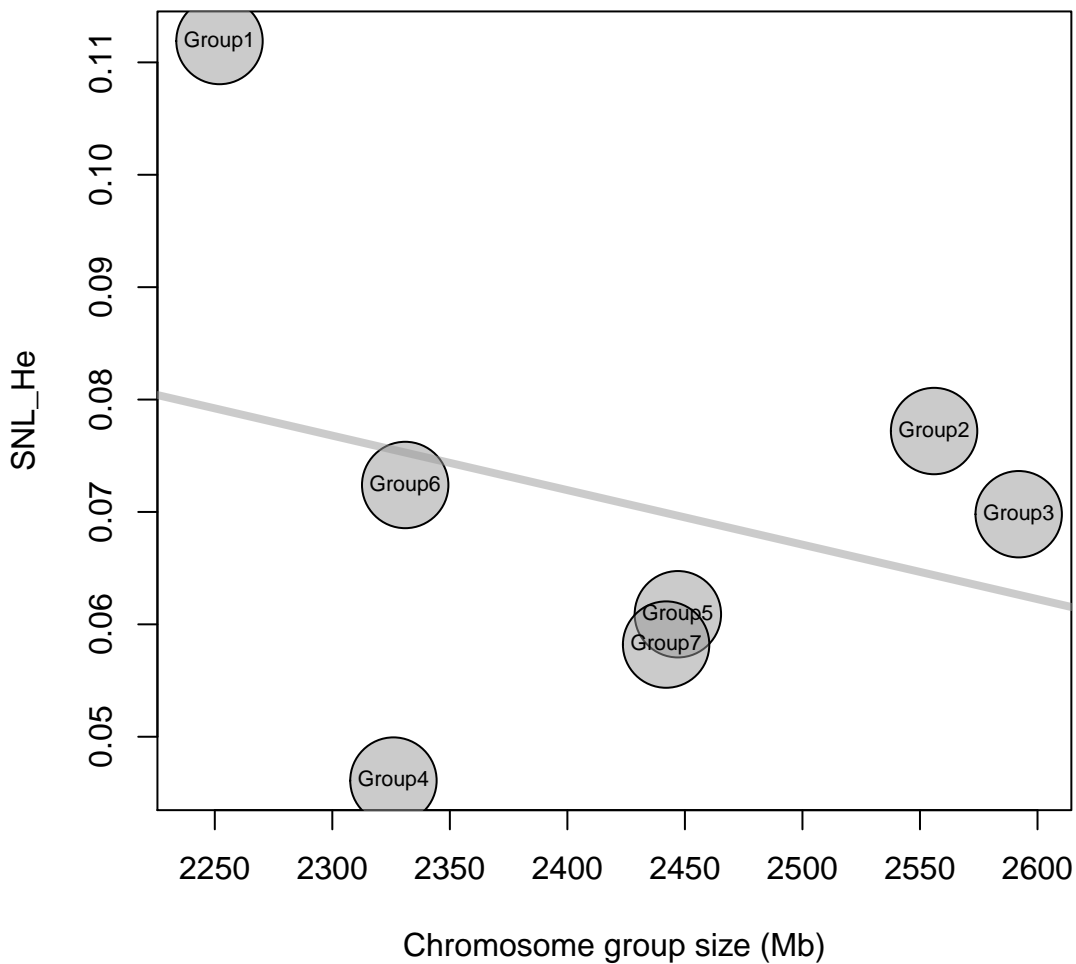

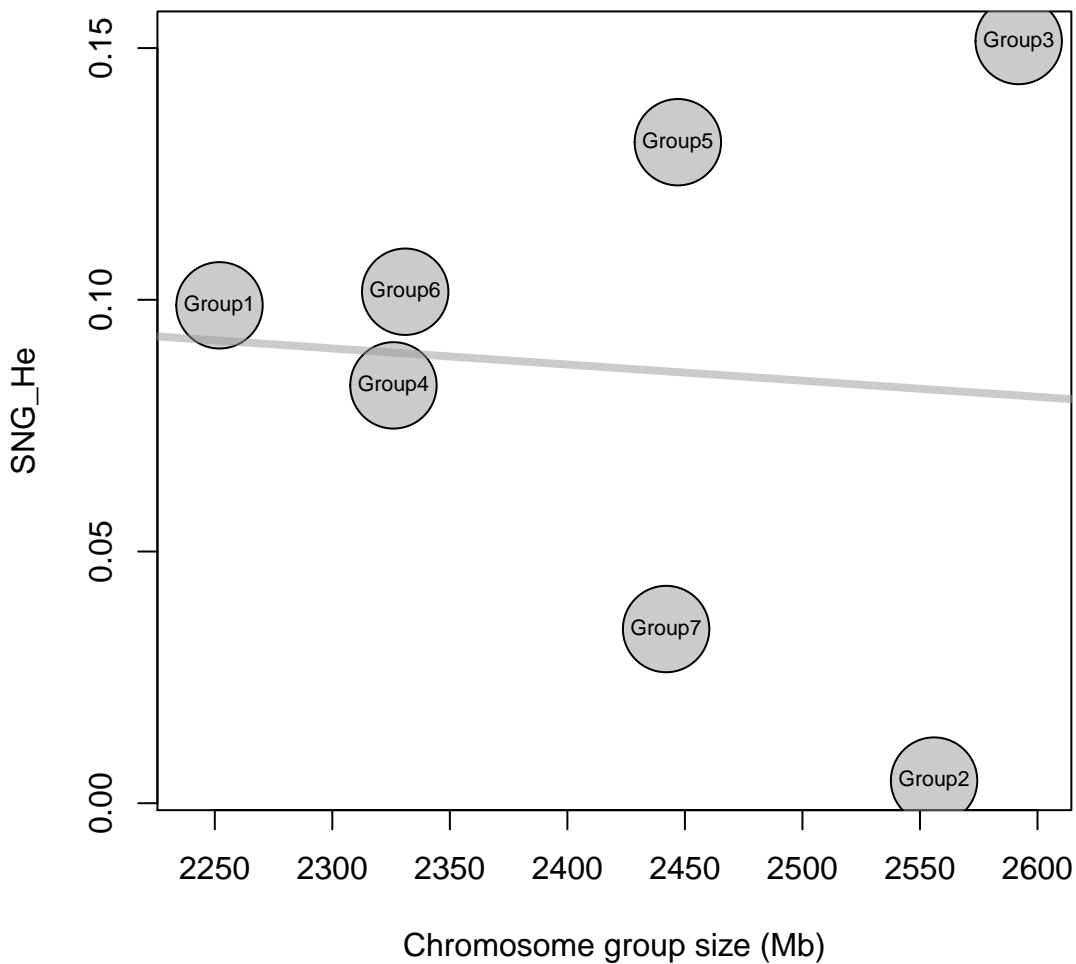

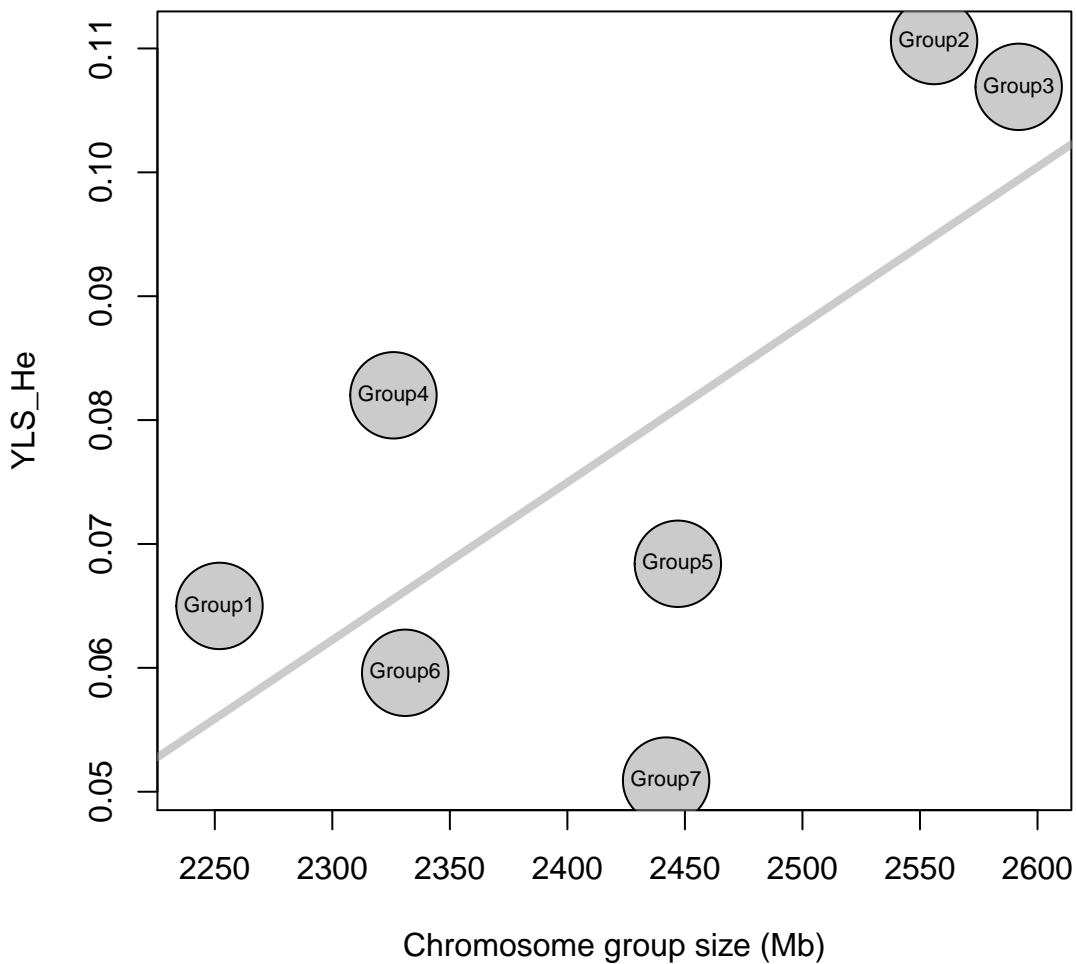

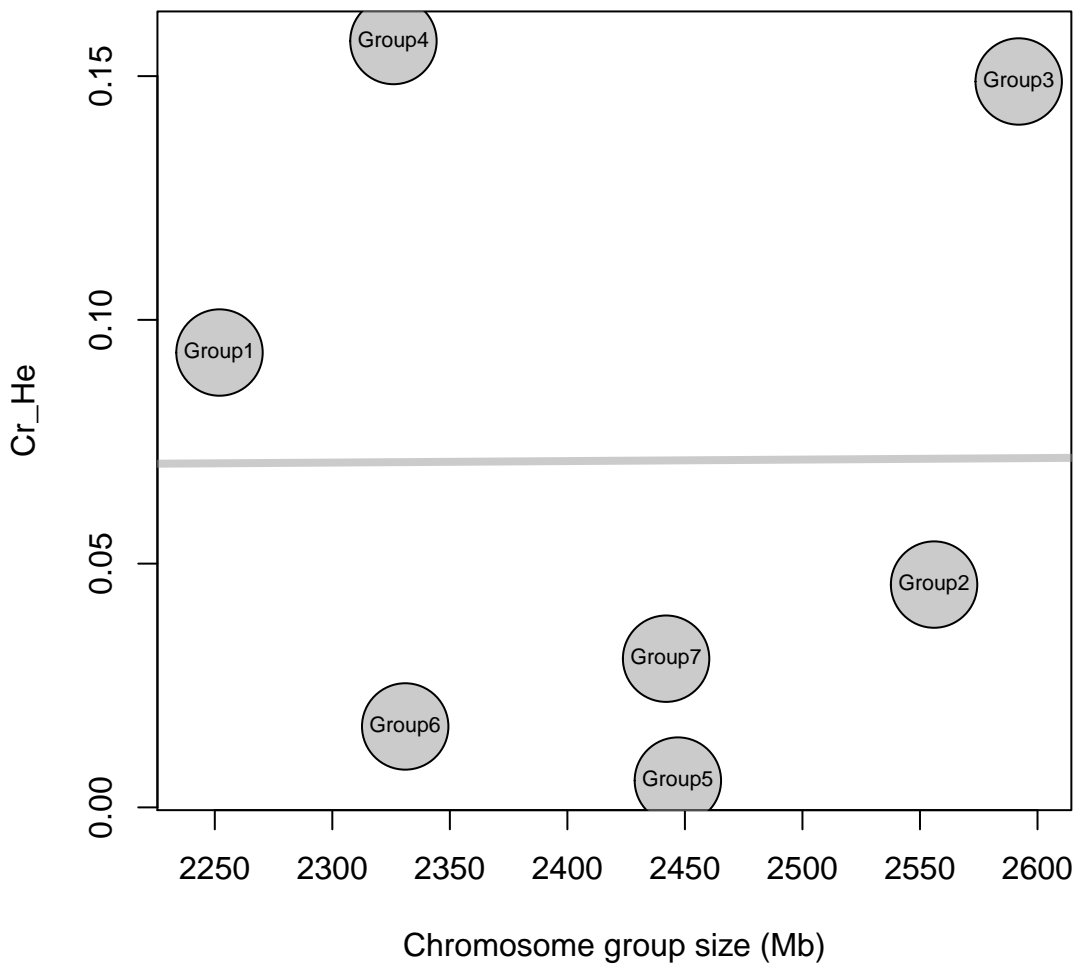

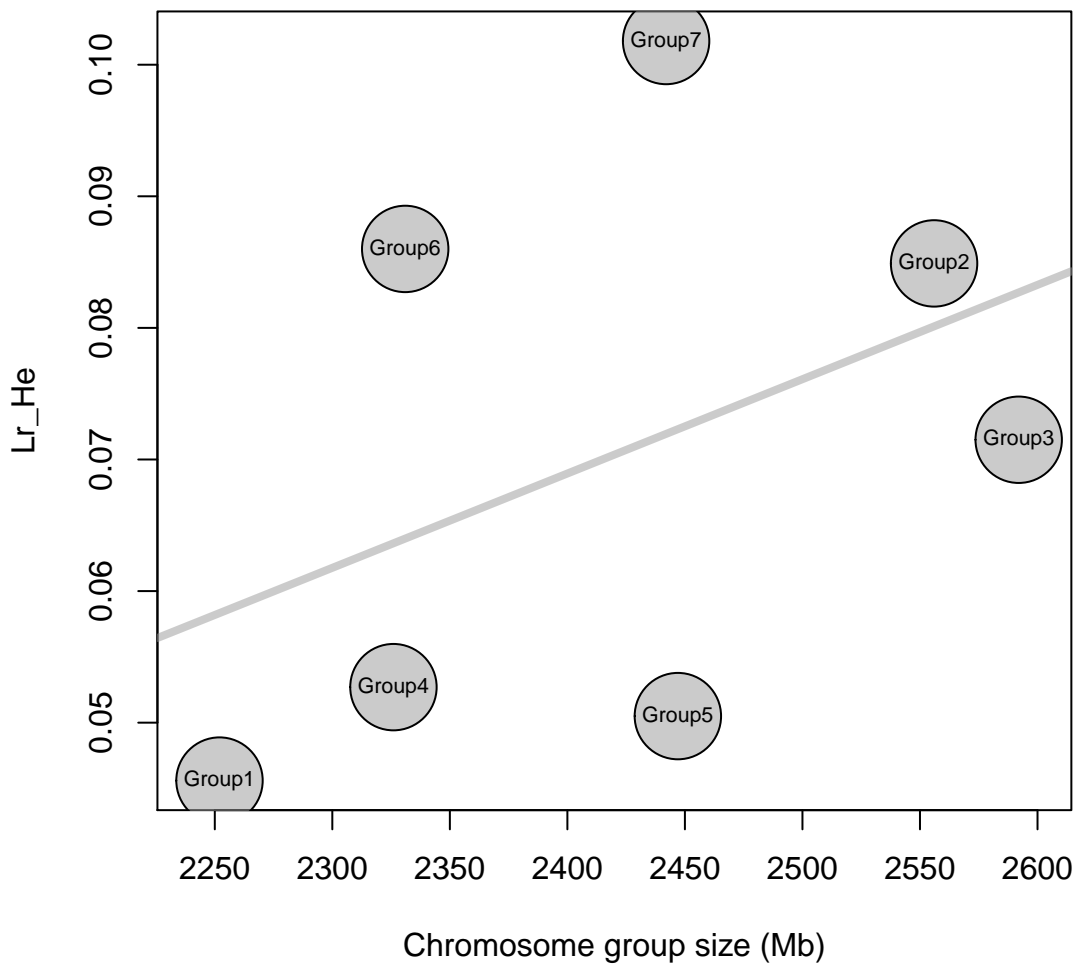

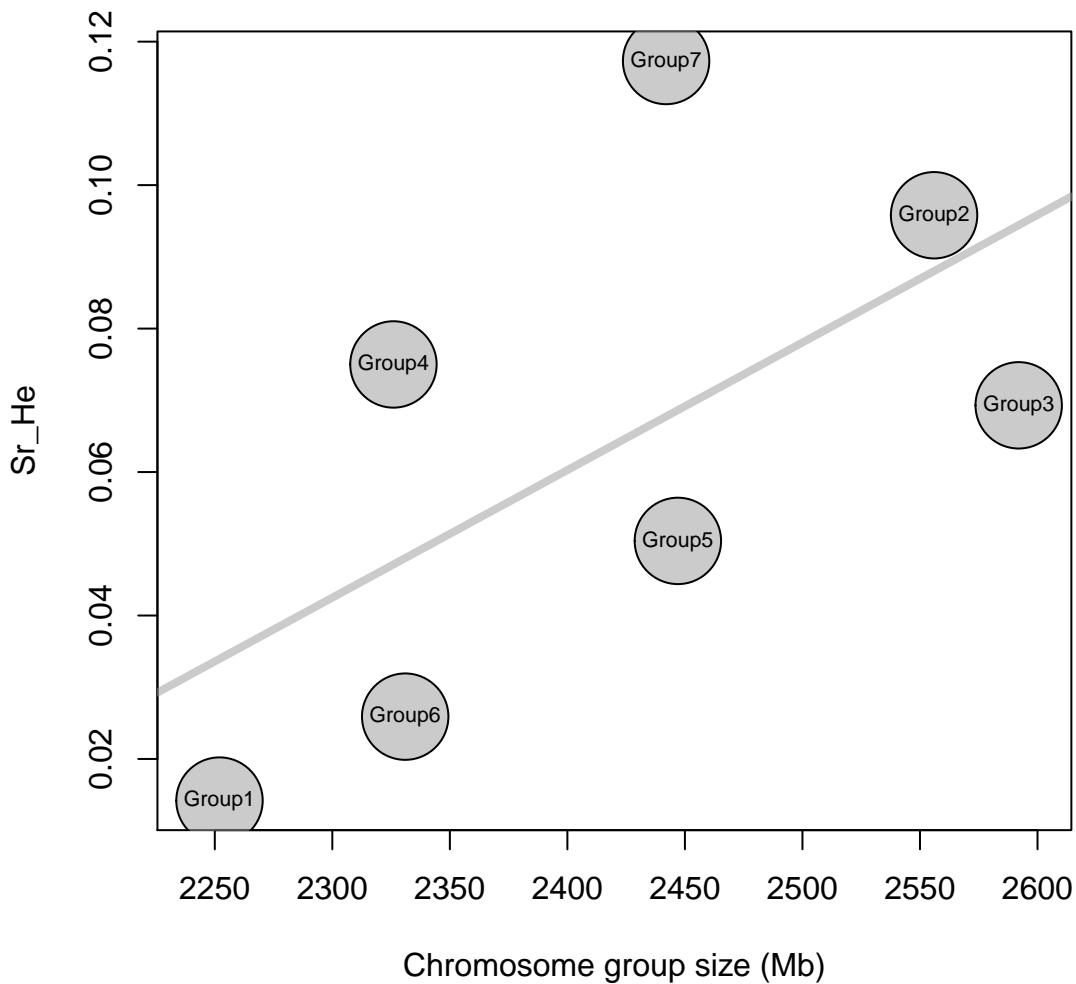

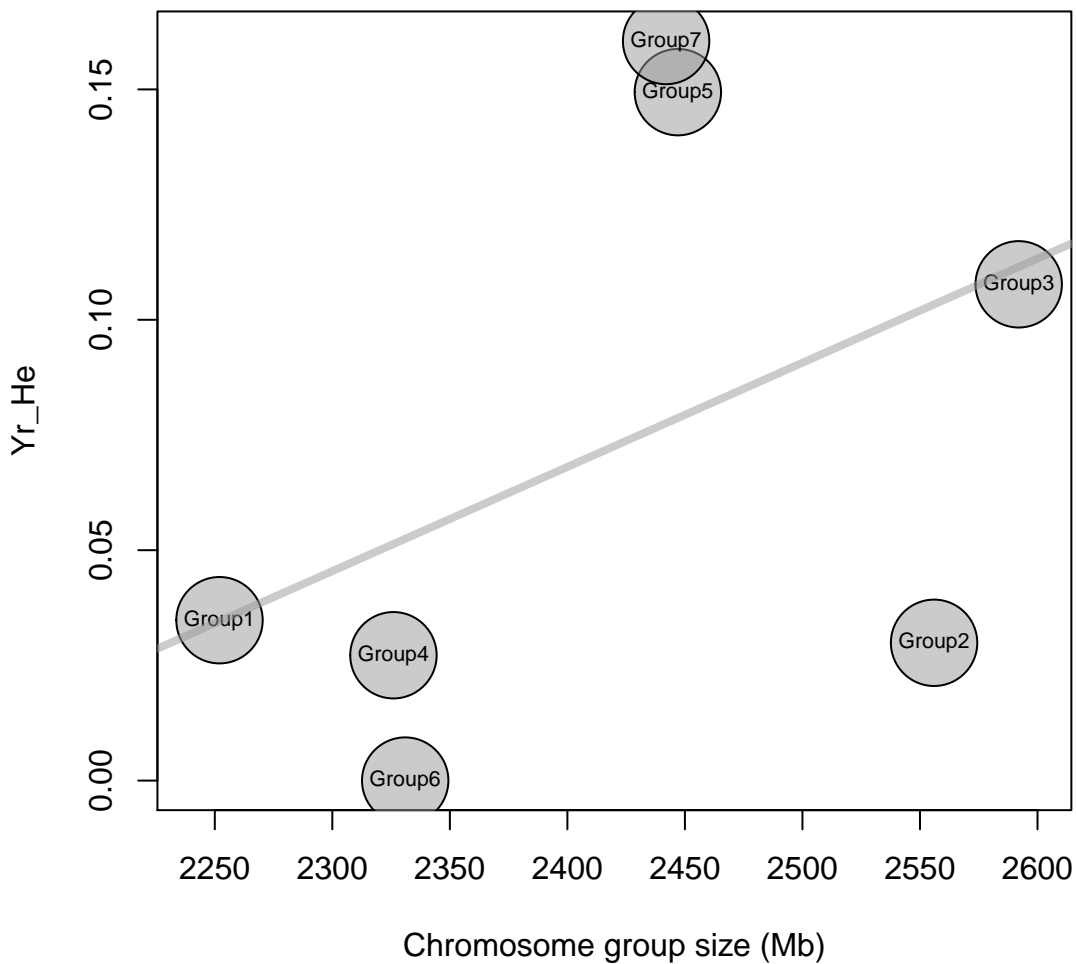

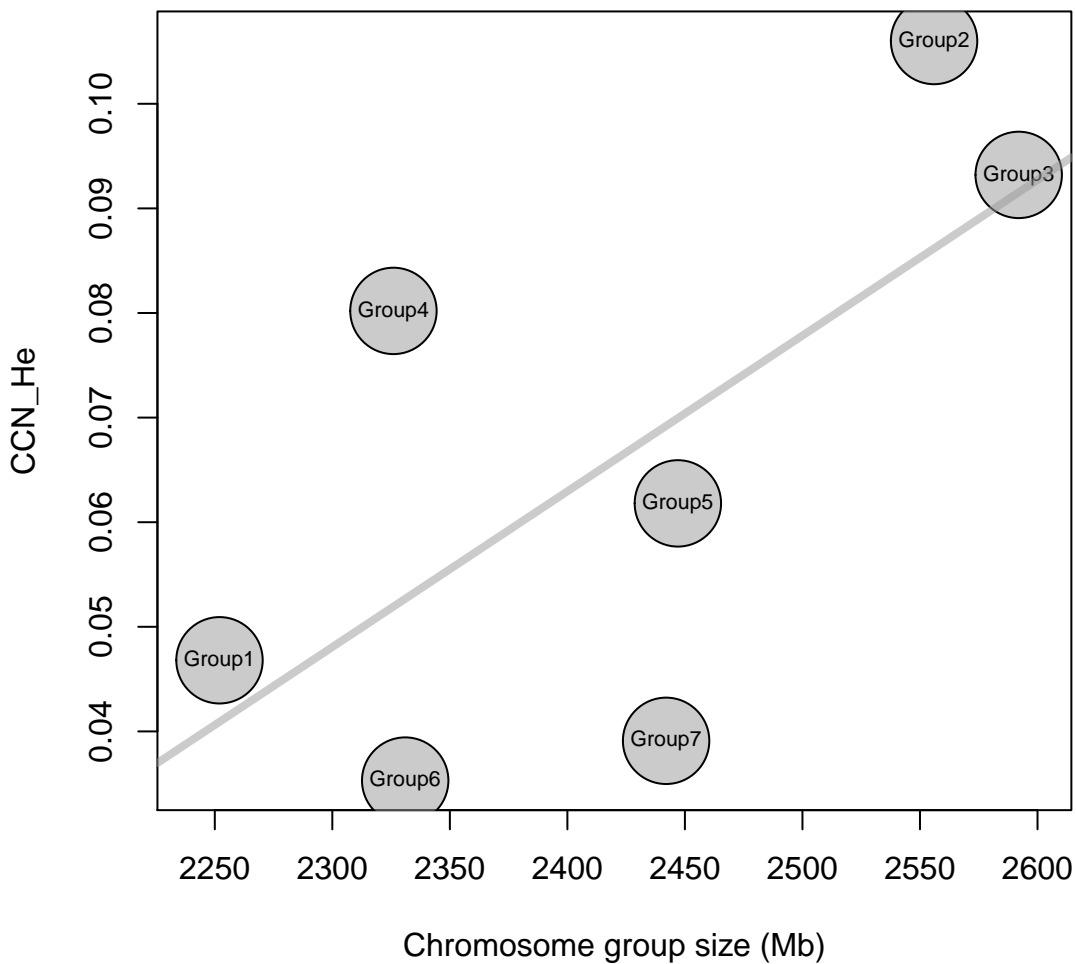

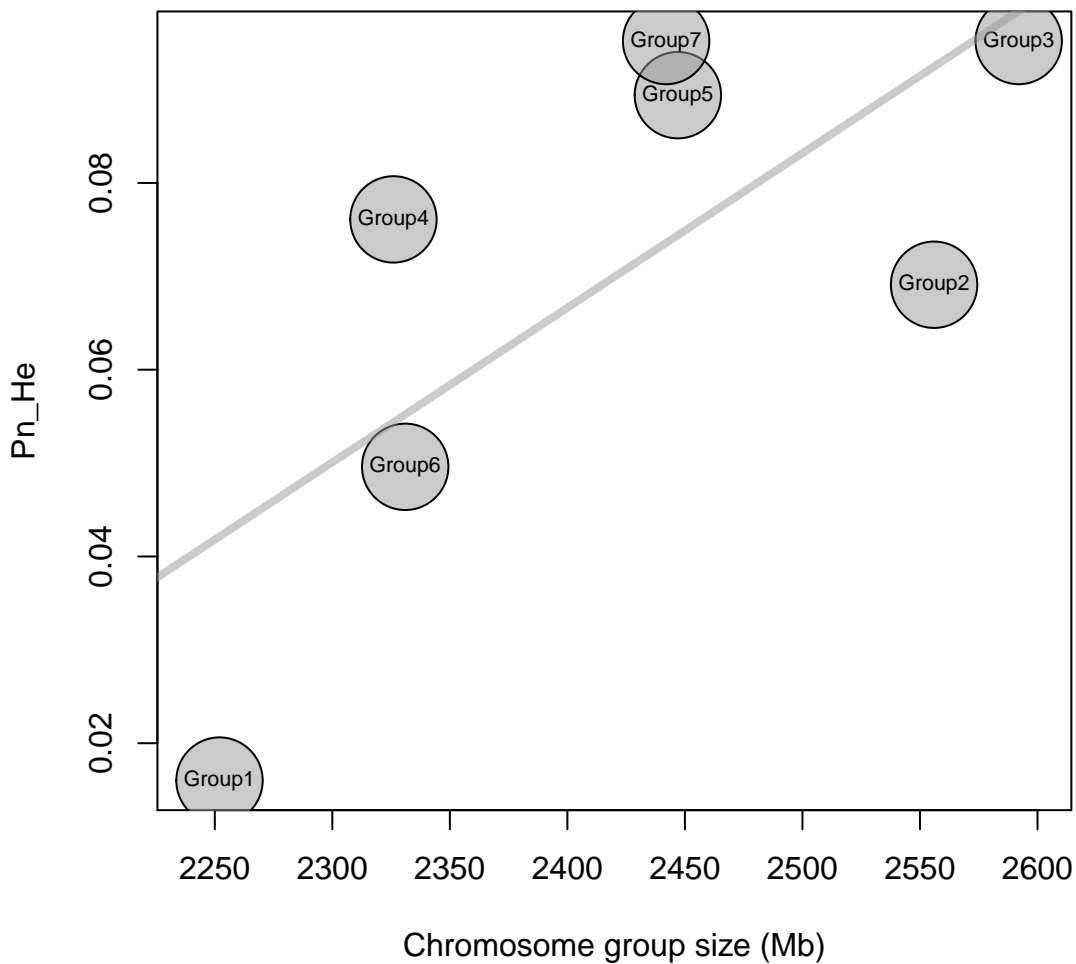

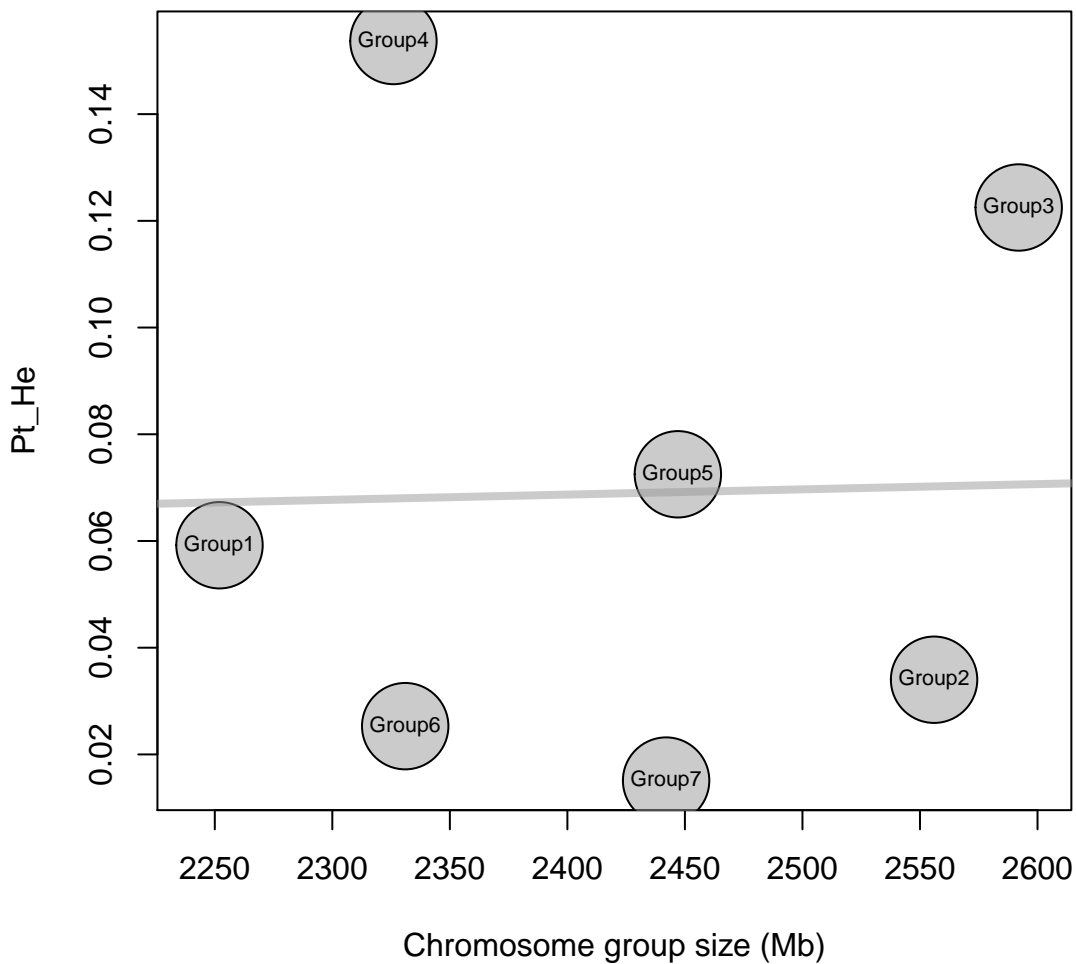

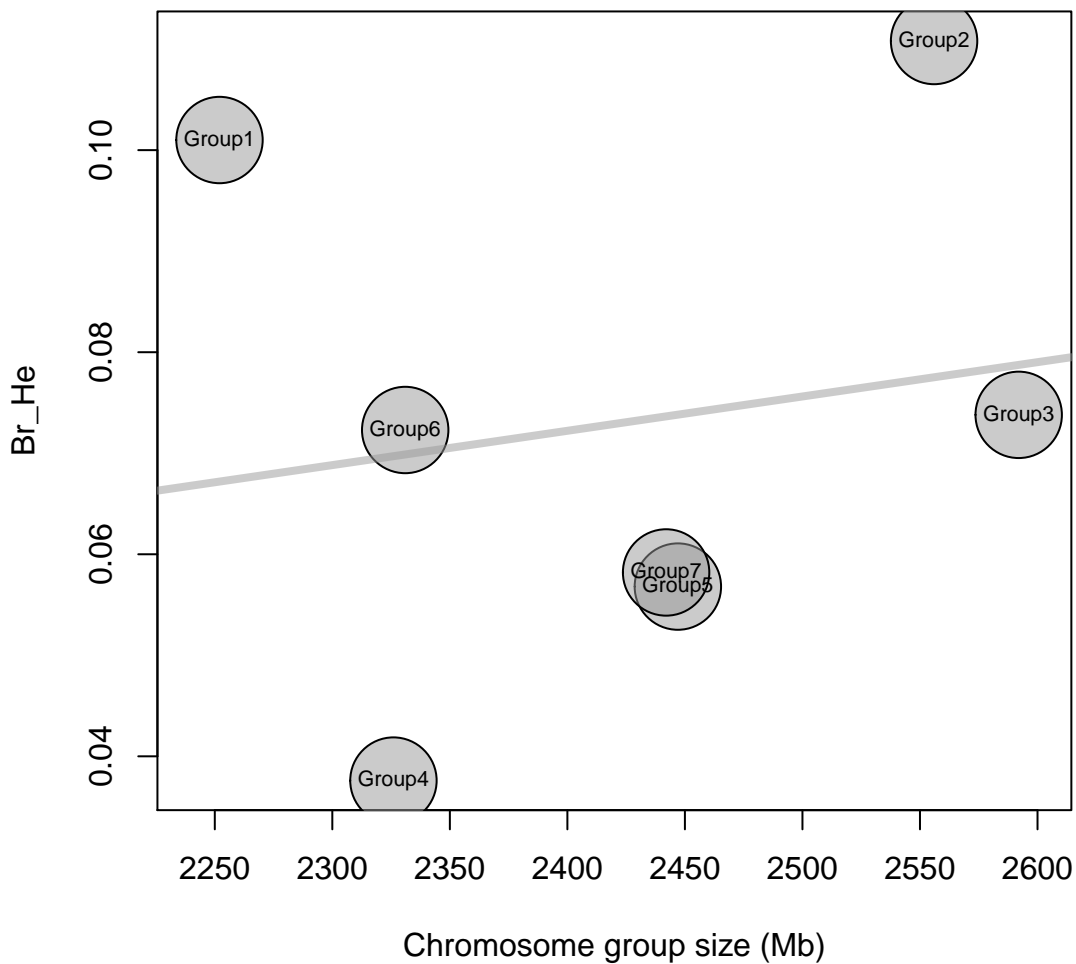

Al<sub>He</sub>

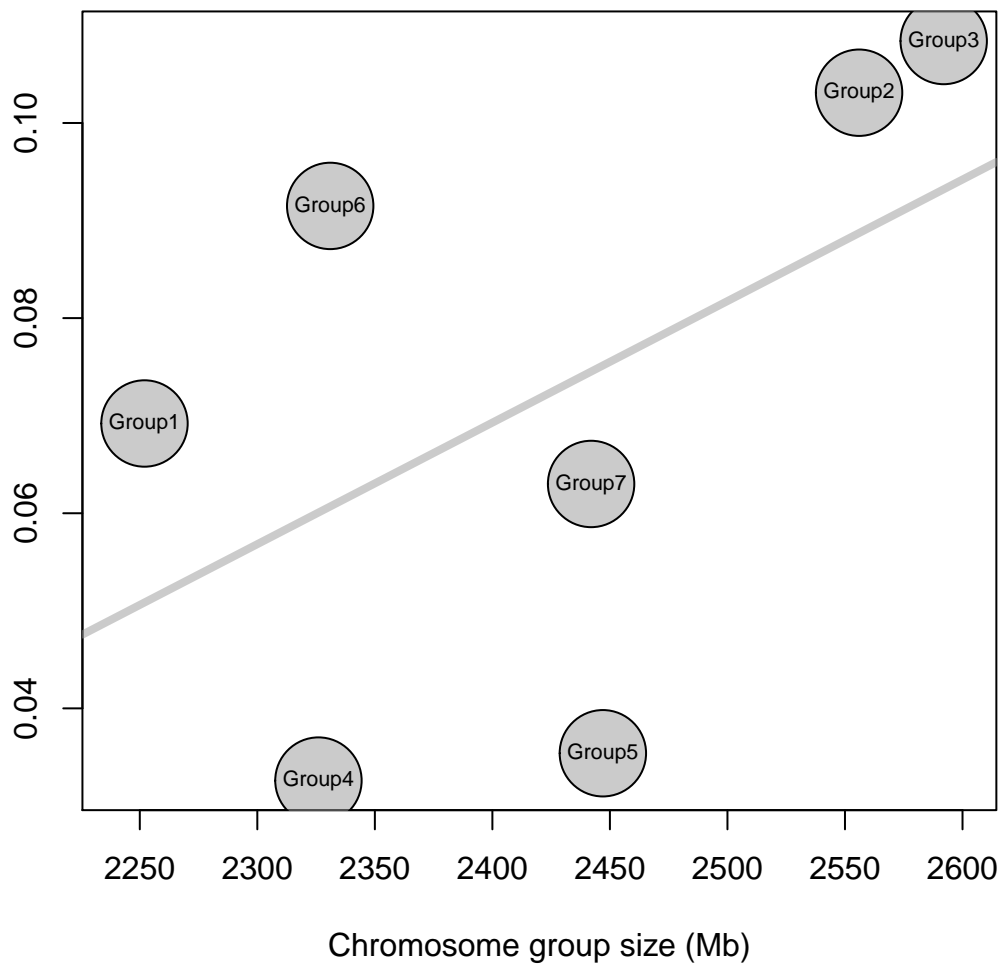

Supplement: Figure S2 — Percentage of chromosomal group contribution to the additive variance for each trait as function to chromosome size. [file Image2.PDF]
